# Selective microRNA uridylation by Zcchc6 (TUT7) and Zcchc11 (TUT4)

**DOI:** 10.1093/nar/gku805

**Published:** 2014-09-15

**Authors:** James E. Thornton, Peng Du, Lili Jing, Ljiljana Sjekloca, Shuibin Lin, Elena Grossi, Piotr Sliz, Leonard I. Zon, Richard I. Gregory

**Affiliations:** 1 Stem Cell Program, Boston Children's Hospital, Boston, MA 02115, USA; 2 Department of Biological Chemistry and Molecular Pharmacology, Harvard Medical School, Boston, MA 02115, USA; 3 Department of Pediatrics, Harvard Medical School, Boston, MA 02115, USA; 4 Harvard Stem Cell Institute, Cambridge, MA 02138, USA; 5 Department of Genetics, Harvard Medical School, Boston, MA 02115, USA; 6 Howard Hughes Medical Institute, Boston, MA 02115, USA

## Abstract

Recent small RNA sequencing data has uncovered 3′ end modification of mature microRNAs (miRNAs). This non-templated nucleotide addition can impact miRNA gene regulatory networks through the control of miRNA stability or by interfering with the repression of target mRNAs. The miRNA modifying enzymes responsible for this regulation remain largely uncharacterized. Here we describe the ability for two related terminal uridyl transferases (TUTases), Zcchc6 (TUT7) and Zcchc11 (TUT4), to 3′ mono-uridylate a specific subset of miRNAs involved in cell differentiation and Homeobox (Hox) gene control. Zcchc6/11 selectively uridylates these miRNAs *in vitro*, and we biochemically define a bipartite sequence motif that is necessary and sufficient to confer Zcchc6/11 catalyzed uridylation. Depletion of these TUTases in cultured cells causes the selective loss of 3′ mono-uridylation of many of the same miRNAs. Upon TUTase-dependent loss of uridylation, we observe a concomitant increase in non-templated 3′ mono-adenylation. Furthermore, TUTase inhibition in Zebrafish embryos causes developmental defects and aberrant Hox expression. Our results uncover the molecular basis for selective miRNA mono-uridylation by Zcchc6/11, highlight the precise control of different 3′ miRNA modifications in cells and have implications for miRNA and Hox gene regulation during development.

## INTRODUCTION

MicroRNAs (miRNAs) are small non-coding RNAs that negatively regulate gene expression by binding to complementary sites in the 3′ untranslated regions (3′UTRs) of messenger RNAs (mRNAs) and induce gene silencing via the RNA-induced silencing complex (RISC) (1). While the canonical pathway of miRNA biogenesis is largely understood, there are numerous modifications made to nascent miRNA species that impact their expression and function (2,3). Many of these modifications are appreciated in their ability to enhance or disrupt miRNA processing and repressive ability, while others are only recently being described and characterized (3–5). Recently, several groups have uncovered the importance of widespread modification of 3′ ends of mature miRNAs and shown that this activity occurs for diverse miRNA species depending on the cell line, tissue and developmental stage examined (4–12). Although the enzymes responsible for miRNA 3′ nucleotide addition have in a few cases been identified–and their activities elucidated–much remains unknown about the nucleotide transferases involved in miRNA modification. In nearly every instance of mature miRNA modification only a subset of miRNAs are modified, but thus far no mechanism has been described to explain the underlying specificity of these reactions. Given that much of this activity is restricted to certain cellular contexts, the selective mechanisms of mature miRNA modification may play important roles in development and tissue specification.

RNA-dependent nucleotidal transferases belong to the DNA polymerase ß superfamily of enzymes and include the mRNA Poly(A) Polymerases (PAPs), CCA-adding enzymes responsible for adding the 3′ termini to transfer RNAs (tRNAs), and terminal uridyl transferases (TUTases) or poly(U) polymerases (PAPs) among others (13,14). There are seven mammalian RNA-dependent nucleotidyl transferases including Zcchc11 and Zcchc6 (TUTase4/TUT4/PAPD3 and TUTase7/TUT7/PAPD2, respectively), which play roles at several levels of miRNA biogenesis (15–17). Zcchc11 and Zcchc6 were initially identified as regulators of let-7 miRNA biogenesis in embryonic stem cells where they recognize the let-7 precursor RNA (pre-let-7) when it is bound by the small RNA-binding protein Lin28 (16–18). The Lin28/pre-let-7/TUTase ternary complex is sufficient to oligo-uridylate the 3′ end of pre-let-7 and facilitates its degradation by the exonuclease Dis3l2 (19,20). This miRNA turnover pathway serves to keep let-7 miRNAs low in undifferentiated cells and, upon the transition to differentiation, is relieved to ultimately permit the accumulation of mature let-7 miRNAs in differentiated cells (16–18,21–23). Zcchc11 and Zcchc6 along with GLD2/PAPD4, a TUTase previously shown to have mono- and oligo(A)-adding activity, together can add single uridine residues to the 3′ end of certain precursor let-7 miRNAs to create a 3′ end structure that facilitates efficient Dicer processing (15). This activity is Lin28-independent and is thought to occur constitutively in differentiated cells. Interestingly Zcchc11 was independently shown to uridylate the 3′ end of mature miR-26 and thereby attenuate its activity toward target mRNAs involved in the inflammatory response (6). The repertoire of Zcchc11-targeted miRNAs was recently expanded to include a subset of mature miRNAs involved in growth hormone regulation and deletion of Zcchc11 disrupted normal growth patterns in fetal mice (8). It remains unclear how mono-uridylation blocks miRNA target repressive ability or how miR-26 and the several other miRNAs targeted by Zcchc11 and Zcchc6 are specified, but mature miRNA uridylation consistently fails to alter the steady-state levels of mature miRNAs and instead likely acts through changes in target specificity, RISC loading or other mechanisms (8).

To further understand the role of Zcchc11 and Zcchc6 mature miRNA uridylation, we investigated whether these two TUTases have an inherent preference toward various miRNA substrates and found that a simple sequence motif exists that is necessary and sufficient for targeting by Zcchc11 and Zcchc6 *in vitro*. Intriguingly, the only miRNAs that contain this motif are known regulators of developmental genes, and in several cases are highly enriched for targeting Hox gene 3′UTRs. The predicted miRNAs found in our search comprise four families, specifically: let-7, miR-99/100, miR-196a/b and miR-10a/b family members. In cultured cells, the depletion of Zcchc11 and Zcchc6 led to a significant reduction in 3′ uridylation in a small number of miRNA species, many of which could be predicted by our sequence-based approach. Furthermore, we were surprised to find that after TUTase depletion and a corresponding drop in uridylated mature miRNAs, there was a seemingly compensatory increase in mono-adenylation, effectively maintaining a constant pool of modified miRNAs. These results suggest that miRNA 3′ uridylation is at least partially explained by preferential activity of Zcchc11 and Zcchc6, and that this uridylation may impact the developmental program in animals. There may also be a miRNA PAP that functions in response to the loss of uridylation, and regulates the relative levels of modified versus unmodified miRNAs. Consistent with these findings, inhibition of the orthologous genes in Zebrafish results in improper *Hox* gene expression and developmental phenotypes.

## MATERIALS AND METHODS

### Uridylation assays

*In vitro* uridylation assays were performed as described previously, except with a final RNA concentration of 300 nM (18). Synthetic RNAs were purchased from IDT.

**Table:** 

RNA oligo	Sequence
let-7g guide	UGAGGUAGUAGUUUGUACAGUU
let-7g passenger	CUGUACAGGCCACUGCCUUGC
GL2 guide	UCGAAGUAUUCCGCGUACGUU
GL2 passenger	CGUACGCGGAAUACUUCGAUU
let-7i guide	UGAGGUAGUAGUUUGUGCUGUU
let-7i passenger	CUGCGCAAGCUACUGCCUUGCU
let-7i domains 1/2 mut	UAGUCGCUGCAUUUGUGCUGUU
let-7i domains 2/3 mut	UGAGGUAUGCAGCCUAGCUGUU
let-7i guide 1/2/3 mut	UAGUCGCUGCAGCCUAGCUGUU
let-7i delG	UUACAUACUAAUUUCUACUCUU
GL2 with let-7 motif	UCGAAGUGUAGUUUGUACGUU
miR-10a guide	UACCCUGUAGAUCCGAAUUUGUG
miR-26a guide	UUCAAGUAAUCCAGGAUAGGC
miR-10a double mut	UACCCUUGCAAUCCGAAGCCUAG
let-7g+A guide	UGAGGUAGUAGUUUGUACAGUUA
let-7g+U guide	UGAGGUAGUAGUUUGUACAGUUU

### Expression plasmids and recombinant Zcchc11 purification

Human Zcchc11 cDNA region encoding amino acids 225–1384 was subcloned into a modiﬁed pET-24 plasmid (Novagen) containing an N-terminal His6-Trx (thioredoxin) tag with a cleavage sequence for TEV (tobacco etch virus) protease. Protein expression in *Escherichia coli* BL21 (DE3) STAR strain was induced with 1 mM IPTG (isopropylβ-D-thiogalactoside) for 5 h at 25°C. Large-scale purification of the recombinant protein was performed as follows. Cells were harvested and resuspended in 50 mM Hepes (pH 8.0), 150 mM NaCl, 10 mM imidazole, 0.2% Igepal CA-630 (Sigma-Aldrich), 0.5 mM 2-mercaptoethanol and 1 mM PMSF, supplemented with the Complete EDTA-free protease inhibitors mix (Roche), and lysed by freeze-thawing and ultrasound sonication. The soluble fraction of centrifugation-clariﬁed cell lysate was applied on to Ni-NTA (Ni^2+−^nitrilotriacetate) metal-afﬁnity chromatography matrix (Qiagen) and washed with high salt containing wash buffer (20 mM Hepes-NaOH, 1M NaCl, 20 mM imidazole, 1 mM TCEP); the recombinant His6-Trx-tagged protein were eluted with 50 mM Hepes/NaOH (pH 8.0), 300 mM imidazole and 0.5 mM 2-mercaptoethanol and dialysed into 50 mM Hepes/NaOH (pH 8.0), 150 mM NaCl, 20 mM imidazole, 0.5 mM 2-mercaptoethanol. The His6-Trx tag was cleaved off by digestion with TEV protease at 4°C. The protein was further purified by additional passage on Ni-NTA, to separate it from the His-TRX tag, and exchanged in the final buffer 150 mM NaCl, 20 mM HEPES, pH 7.5, 2 mM MgCl_2_, 5% glycerol and 1 mM TCEP. Recombinant Zcchc11 Nt-Ct was quantified by comparison with the known amounts of BSA on SDS-PAGE gel.

### RNA electromobility shift assay (EMSA)

EMSAs were conducted using P32 5′-end labeled let-7g guide and passenger probes and 3–50 ng of recombinant human Zcchc11. Binding reactions were conducted in 10 μl total volume. Binding buffer contained 500 mM NaCl, 20 mM HEPES, pH 7.5, 2 mM MgCl_2_, 5% glycerol and 1 mM TCEP. RNA–protein complexes were resolved on native 12% polyacrylamide gels, run at 100 V, in 0.5× TBE.

### Cell culture

P19 cells were cultured in αMEM+10% FBS with daily addition of 100 nM retinoic acid over the time course described.

### Antibodies

The following antibodies were used for western blots in this study: α-Zcchc11 (Proteintech Group #18980-1-AP), α-Zcchc6 (OpenBioystems, custom rabbit polyclonal antibody), α-Flag (Sigma #A8592), α-Lin28A (Cell Signaling #3978), α-Tubulin (Abcam #AB6046), α-Actin (Sigma #A2066).

### Mouse tissue extraction

Tissues and organs were collected from >5-month-old mice and were homogenized. Lysates were cleared and quantitated using Bradford reagent before immunoblotting.

### Lentivirus production and infection

Lentivirus was prepared according to the manufacturer's protocol (Invitrogen #K4975-00) and supernatant was filtered through a 0.45 μm filter before being stored at −80°C or used immediately. HeLa cells were transduced with 500 ul of lentivirus supernatant in the presence of polybrene (4 μg/ml) and incubated overnight. Media containing either Puromycin (shZcchc11, shGFP, 2.5 μg/ml) or Hygromycin (shZcchc6, 140 μg/ml) or both (shZcchc11/6) were added 36-h post-infection and stably resistant cell pools were grown before analysis.

### shRNAs

**Table:** 

Plasmids	Identifier	Targeted sequence	Resistance
pLKO+shGFP	Sigma SHC005		Puro
pLKO+shZcc11 #1	Sigma #TRCN0000150277	TCAGTTACATTCAGCAGAAA	Puro
pLKO+shZcc11 #2	Sigma #TRCN0000146303	CGTGATAGTGATCTGGATATT	Puro
pLKO+shZcchc6 #408	Custom	GGAATTGCTGCGGTTCTATGC	Hygro
pLKO+shZcchc6 #409	Custom	GTGACCTTGACGTCTGTATGA	Hygro

### Small RNA sequencing and bioinformatics analysis

Small RNAs were cloned according to the manufacturer's protocol (Epibio #SMMP101212). Briefly, 50 ug of Trizol-purified total RNA (Invitrogen) was fractionated using miRvana columns (Invitrogen) and 300 ng of <200 nt RNA was used in the cloning reactions. Resulting libraries were gel purified from a NativePage 4–16% Bis-tris acrylamide gel (Life Technologies #BN1002BOX) and subjected to Illumina sequencing. 18–30nt small RNA sequences were used for analysis, after removing the adaptor sequences. Bowtie program (http://bowtie-bio.sourceforge.net/index.shtml) was used for aligning small RNAs to annotated human miRNA sequences in miRBase database (http://www.mirbase.org), and two mismatches were permitted. MicroRNA sequences with one ‘U’ and ‘A’ mismatches at the 3′ end were considered as untemplated uridylated and adenylated miRNA, respectively.

### Zebrafish strain and morpholino (MO) injection

Zebrafish were maintained following animal research guidelines at Boston Children's Hospital. The wildtype strain (Tu) and p53 mutant strain were used in this study. MOs (listed below; GeneTools) were injected into embryos at 1- to 4- cell stage. Standard control morpholinos (GeneTools) were used in the control groups. Predicted partial reference sequences, ENSDARG00000074645 for *zcchc6* and ENSDARG00000070271 for *zcchc11*, were annotated in zebrafish genome (http://www.wensemble.org/Danio_rerio/gene/). TBLASTN analysis using human zcchc6 or zcchc11 protein sequences against various zebrafish nucleotide sequence databases, such as EST, WGS contigs, TSA and nr, identified the same sequences as the annotated partial sequences. Zcchc6 MO-1 was designed to block the splicing of the C2H2 domain containing exon and zcchc6 MO-2 was to block the exon encoding the catalytic domain. Zcchc11 MO-1 and MO-2 were also designed to block the splicing of exons containing C2H2 domain and active domain, respectively.

**Table:** 

Morpholino name	Sequence	Dose (ng/embryo)
zcchc6 MO	GTGAGTTTGTGTCCAGTTACCTTGA	1
zcchc6 MO-2	TGTATGTGTTTAGACTCACAGTGGA	8
zcchc11 MO	TGGCCTTCTAAAGTCATGCAGATGT	8
zcchc11 MO-2	TGTGCGGTTACACACCTGGAAGAAC	2.5

### *In situ* hybridization

*In situ* hybridization was performed as previously described (24). The images were processed using Adobe Photoshop.

### Reverse transcription PCR (RT-PCR) and quantitative reverse transcriptase PCR (q.RT-PCR)

For RT-PCR analysis following morpholino injection, RNA from 20 pooled embryos was extracted and after cDNA synthesis, PCR using gene-specific primers was performed (primer sequences listed below).

**Table:** 

Target transcript	Forward	Reverse
zcchc6MO	ACATCTCGCCAGCTCTCAGT	GCACACAACTACAGGCATCC
zcchc6MO-2	CAAAGTTGGTTTTATTTGGCTCA	CTTCACAATTGGGACTTTCG
zcchc11MO	GTGTTGGAGGCAGCAGAACT	CGCGAGGTGATTGGTAGTG
zcchc11MO-2	CAAAGCGTCATCCTCCAAAC	TAATTCCCTCCATCCGAAGC

q.RT-PCR was performed on cDNA isolated from the 20 embryos. All primer sequences are listed.

**Table:** 

Target hox transcript	Forward	Reverse
*a1a*	TGGATGAAGGTTAAACGCAAC	CGAAAAATTGGTGCGTACAG
*a9a*	AATTCCTGCGGAGACGAAG	CACCGCTTTTTCCTAGTGGA
*b3a*	AACAGCTCCCCTAGTGCAAG	GGAGGGCTTTTCTCACCAC
*b5a*	CCCAAATATTCCCTTGGATG	ATAGCGGGTATATGCAGTTCG
*c6a*	AGATCTACCCGTGGATGCAG	TTCCAAGGTTTGGTATCTGGA
*d3a*	AGCAGAAAAGCACCAACTGC	CAGGCGGACTCTTGTCATC
*d10a*	AACTGAGGCGTCTGTTTCCA	CGGTTAACCAGTTGCTCGTC
*b8b*	CCCATGGATGAGACCACAA	CGGGTCAGGTAAGGATTGAA

## RESULTS

### Selective miRNA uridylation by Zcchc11

**Figure 1.**
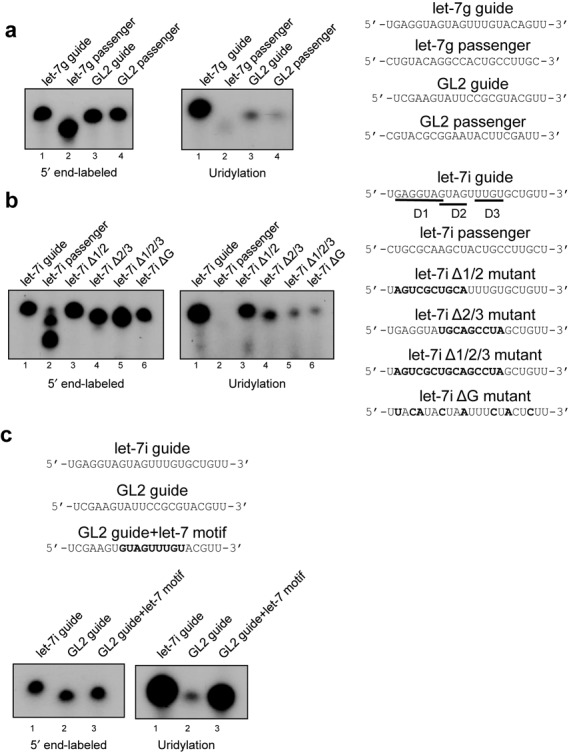
Zcchc11 selectively uridylates mature miRNAs. (**a**) (Left) 5′ end labeling of indicated RNAs shown at right. (Center) Uridylation assay for the same RNA oligos using immunoprecipitated Flag-Zcchc11 purified from 293T cells. (**b**) (Left, Center) 5′ end labeling and uridylation assays, respectively, of indicated RNA oligos shown at right. (Right) Schematic of let-7g guide mutants used in uridylation assay. Bold font indicates mutated residues. (**c**) (Top) RNA oligos used in end-labeling and uridylation assays (below).

Zcchc11 is known to be an important regulator of let-7 maturation in undifferentiated cells, yet its catalytic activity is not restricted to pre-miRNAs. In addition, several mature let-7 miRNAs are subjected to 3′ mono- and oligo-uridylation but the mechanism underlying this activity is poorly understood (4–7,9,15–18). To explore if Zcchc11 has preferential uridylation activity toward specific miRNA substrates, we asked whether mature let-7 miRNAs can be targeted by immunopurified Zcchc11 *in vitro*. Flag-Zcchc11 purified from HEK293T cells was incubated in the presence of single-stranded let-7g guide (let-7g-5p), let-7g passenger (let-7g* or let-7g-3p), or synthetic single-stranded RNAs that target luciferase (GL2 guide and passenger). Using equal amounts of RNA as determined by 5′ end-labeling, we performed *in vitro* uridylation assays and monitored TUTase activity by the incorporation of radiolabeled UTP. We found Zcchc11 preferentially uridylates let-7g guide over let-7g passenger or either strand of luciferase-targeting small RNAs (Figure 1a). We also found Zcchc11 preferentially recognizes single-stranded RNA (ssRNA) over double-stranded RNA (dsRNA) as described previously (6) (data not shown).

The dramatic uridylation preference for one strand of let-7g duplex over the other suggested the presence of specific sequences in let-7g guide that were absent in let-7g passenger. To determine the sequences that convey this substrate specificity, we performed mutagenesis on let-7i guide, another member of the let-7 family. Three contiguous regions of the let-7i guide strand were chosen for further examination; the seed sequence (domain 1), a GXXG motif in the center of the RNA (domain 2) since it is known that CCHC zinc-finger proteins can bind to GXXG motifs (17,25,26) and a U-rich region near the 3′ end of let-7i guide (motif 3). Consistent with the preference shown toward let-7g, let-7i guide was subjected to stronger uridylation activity than let-7i passenger, whereas individually mutagenized RNA for each of the three domains showed no remarkable reduction in uridylation (Figure 1b and data not shown). Interestingly, when combinatorial mutations were made in domains 2 and 3 (Δ2/3), uridylation was more substantially compromised than mutations in domains 1 and 2 (Δ1/3). Mutations in all three domains (Δ1/2/3) showed a reduction in uridylation similar to the domain 2/3 double mutant, implying that this region is necessary for selective uridylation. Finally, a let-7i RNA oligo lacking all guanosine residues (ΔG) was tested, since guanosine-rich sequences are known to mediate the binding of zinc finger–RNA interactions (17,25,26). This mutant also led to compromised activity, similar in extent to the domain 2/3 double mutant and the triple mutant, suggesting that guanosine residues play a critical role in Zcchc11 substrate recognition.

To examine whether domains 2 and 3 of let-7i are sufficient for driving enhanced uridylation, a synthetic RNA designed to target luciferase was modified to contain these regions. Uridylation activity toward GL2 was much weaker compared to let-7i, but was almost completely restored when the let-7 motif was inserted into the GL2 RNA oligo (Figure 1c). These data suggest that specific sequences found in several let-7 miRNAs are both necessary and sufficient to drive preferential uridylation activity of Zcchc11.

### The sequence motif conferring Zcchc11-catalyzed uridylation is present in a small subset of miRNAs

**Figure 2.**
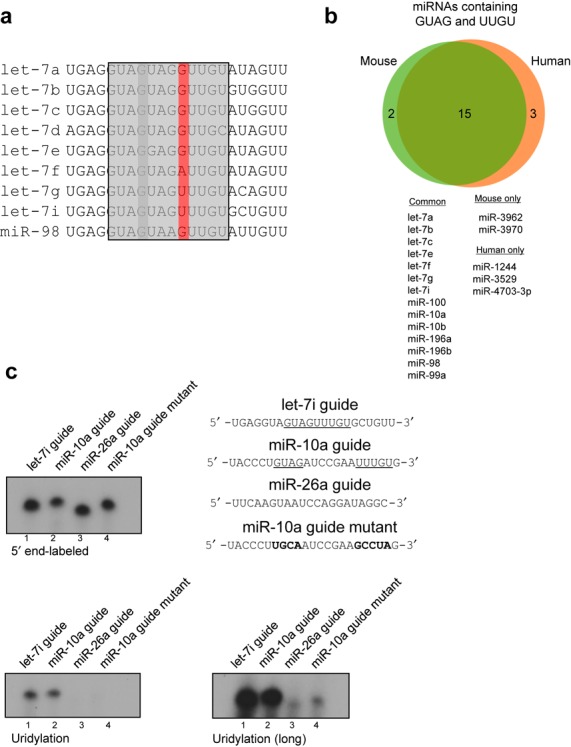
A sequence motif in mature let-7 miRNAs defines Zcchc11 substrates. (**a**) Alignment of mammalian let-7 miRNAs identifies a poorly conserved uridine residue (red) as part of the let-7 motif sequence (gray boxes) that may be dispensable for mature let-7 uridylation. (**b**) Sequence-based search of mouse and human genomes for miRNAs containing GUAG followed by UUGU, with numbers indicating mouse-specific (left circle), human-specific (right circle) or common (overlap) miRNAs. Below, list of identified miRNAs. (**c**) 5′ end-labeling (top left) of RNA oligos as listed (top right). Below, uridylation assay for these miRNAs after either short (left) or long (right) exposure. Underlined residues indicate predicted sequence motif, bold residue indicates mutated regions.

Several mature miRNAs have recently been described that contain non-templated 3′ U residues (5–8), with a limited number undergoing a reduction in uridylation after Zcchc11 depletion (7,8). To gain insight into the mechanism of substrate selectivity and to identify other miRNAs that contain a similar uridylation signal, we performed an alignment of all members of the human let-7 miRNA family. As shown in Figure 2a, all let-7 members contain at least one GUAG sequence, while all except let-7e and miR-98 contain two overlapping GUAG motifs. Surprisingly, however, only let-7i and let-7g contain the UUUGU motif identified above. The first nucleotide in this sequence (shaded dark) is poorly conserved relative to the remaining sequences shared by let-7 miRNAs. We predicted that motifs involved in mature miRNA regulation should be found in all or most members of a given miRNA family, and considered that the domain 2/3 motif may be too stringent and result in false negatives. In light of this, we performed further bioinformatic analyses to determine miRNAs that contain the more limited motif of GUAG followed by UUGU. The reduced stringency of this search could potentially introduce sampling bias and lead us to overestimate putative Zcchc11 target miRNAs, so we compared the lists of miRNAs containing these two sequences in mouse and human databases to observe whether we were identifying a large number of species-specific miRNAs. Importantly, nearly every miRNA identified in this search is found in both mouse (15/17) and human (15/18) databases, while those miRNAs restricted to one species or the other have been identified exclusively in high-throughput sequencing experiments, have no known biological function and often lack homologs in closely related species (Figure 2b).

To determine if the set of putative Zcchc11 substrate miRNAs predicted above are preferred *in vitro*, we carried out uridylation assays using Flag-Zcchc11 and a synthetic RNA for one of these candidates, miR-10a guide (miR-10a-5p). Comparing the activity of Zcchc11 toward let-7i guide, a similar level of uridylation was observed for miR-10a but was lost when the GUAG and UUUGU motifs were mutated to random nucleotides (Figure 2c, lower panel). We also tested the intrinsic preference of Zcchc11 toward miR-26a, a miRNA with reported Zcchc11-dependent uridylation in cells, but found that it did not support strong uridylation similar to let-7 or miR-10 (6). Overall these data support that the identified domains (GUAG and UUGU) are sufficient to predict selective uridylation by Zcchc11 *in vitro*.

**Figure 3.**
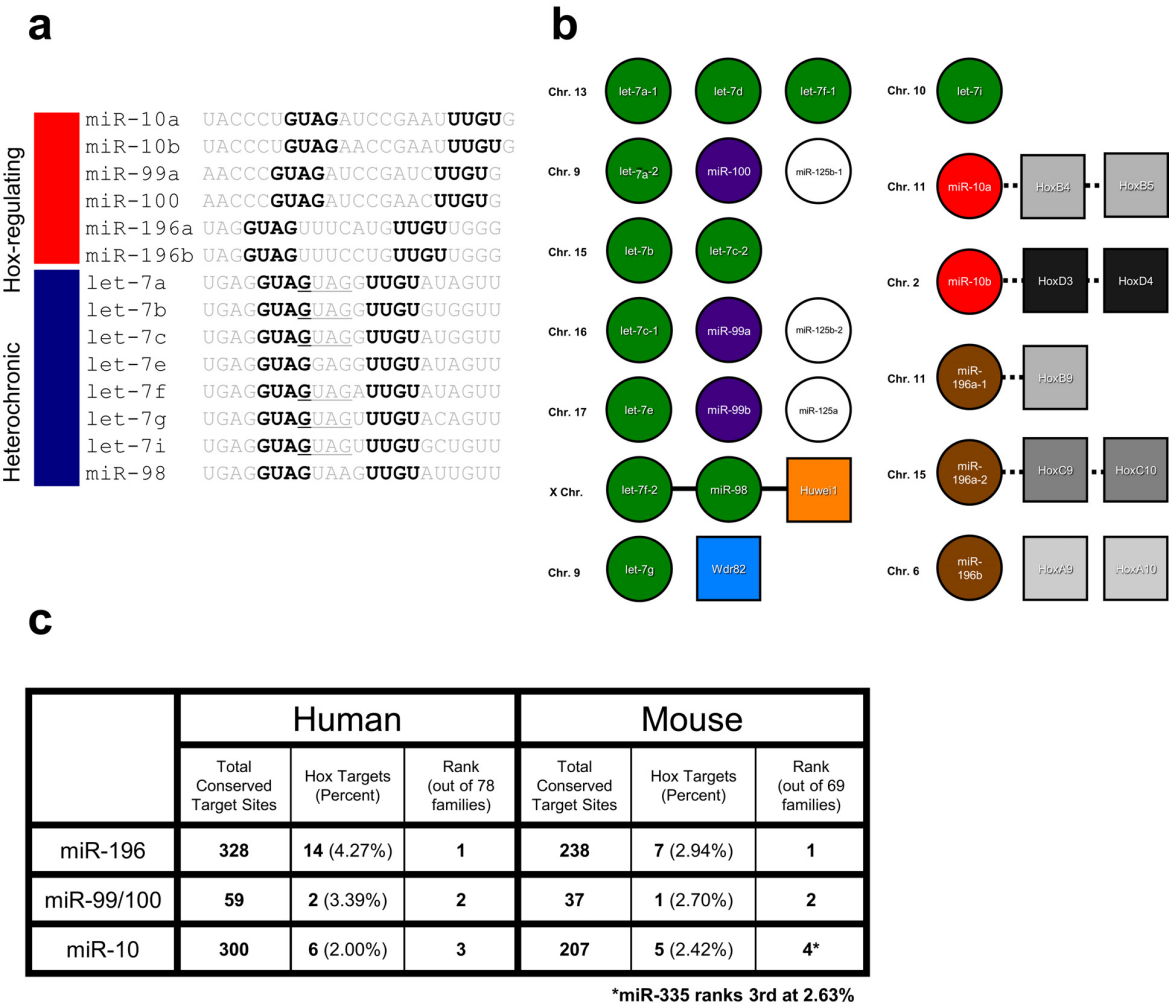
Zcchc11 motif containing miRNAs are involved in development. (**a**) Schematic representation of predicted Zcchc11 substrate miRNAs, classified as either Hox gene-regulators or heterochronic/let-7 family members. At right, alignment of predicted miRNAs with bold and underlined residues indicating conserved sequence motifs. (**b**) Many of the miRNAs predicted to have Zcchc11-targeting sequences are co-expressed in transcriptional clusters or from within Hox gene loci. Each group in a row represents an established or presumed transcriptional miRNA cluster. Solid lines represent miRNAs expressed from introns of coding genes, shown as squares, while dotted lines represent proximal linkage that may or may not indicate co-transcriptional coupling. Chromosomal location indicated at left of each column. (**c**) Table showing frequency and rank of indicated Hox-targeting miRNAs in both mouse and human genomes.

Examining the list of predicted Zcchc11 miRNA substrates led to a striking observation; all of the miRNAs in this analysis fall into just four families (Figure 3a). Roughly half of this group, the let-7 miRNA family, is the best-characterized heterochronic miRNA family, known to regulate developmental timing in organisms as diverse as *C. elegans* and humans (27,28). The remaining miRNAs found in our search prediction are known regulators of development, functioning primarily through repressing Homeobox (Hox) genes during posterior-anterior patterning in vertebrates (29). The three families we identified, miR-10, miR-99/100 and miR-196, are highly conserved and are dysregulated in a number of human diseases. Intriguingly, many of these miRNAs are expressed from the same genomic clusters and are often encoded within various Hox loci (Figure 3b). To examine the relative importance of these three miRNA families in Hox gene regulation, we asked which miRNA families most frequently target the 3′UTR of Hox gene mRNAs. In both human and mouse genomes, these three miRNA families are some of the most likely to target Hox gene mRNAs, and several Hox gene UTRs contain multiple sites for several different members of these miRNA families (Figure 3c and data not shown). For the various members of the predicted miRNA families, this sequence-based approach identified all members except for let-7d and miR-99b. In both instances these miRNAs were not predicted based on a U-to-C transposition at the final position of UUGU. The enrichment of Hox-targeting and heterochronic miRNAs suggests that Zcchc11 may specifically target developmentally important miRNAs.

### Selective miRNA uridylation by an alternative TUTase and recombinant Zcchc11

**Figure 4.**
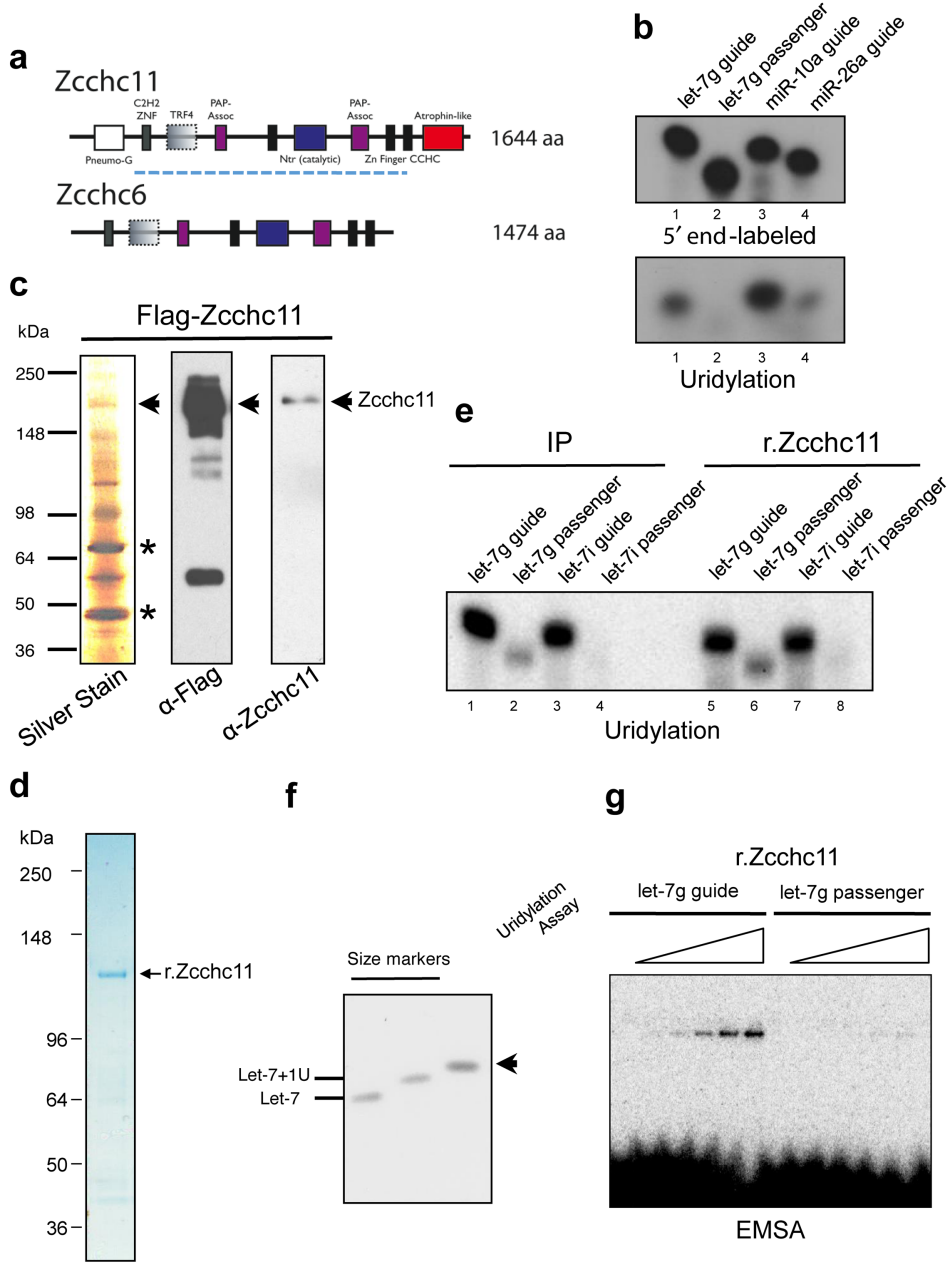
Selective miRNA uridylation by an alternative TUTase and recombinant Zcchc11. (**a**) Schematic representation of mouse Zcchc6 and Zcchc11 proteins. Dashed line indicates the domains included in the recombinant Zcchc11 (r.Zcchc11) used in d-g. (**b**) Zcchc6 targets miRNAs with similar preference as Zcchc11. End-labeling and uridylation assays as in Figure 1. (**c**) Analysis of Flag-affinity-purified Zcchc11-containing complex(es) by silver staining and western blot with the indicated antibodies. Asterisks denote the common contaminants SKB1 and MEP50. (**d**) Analysis of bacterially expressed His-Zcchc11 (r.Zcchc11) by Coomassie blue staining. (**e**) Uridylation assays performed with Flag-purified or recombinant Zcchc11 and the indicated RNA substrates (Left). The recombinant Zcchc11 protein (amino acids 225–1384) lacks N-terminal pneumoG motif as well as the C-terminal atrophin-like region. (**f**) Single-nucleotide resolution analysis of miRNA uridylation assays. Reaction products with r.Zcchc11 were resolved on denaturing 15% PAGE and the indicated synthetic 5′ end-labeled RNAs were used as size markers. A single band that corresponds to the mature miRNA with a two-nucleotide extension was observed. (**g**) EMSA with the indicated radiolabeled RNA probes (2.5 nM final concentration) and increasing amounts (1x, 2x, 4x, 8x and 16x) of recombinant Zcchc11 protein.

Zcchc6 is a TUTase that shares significant homology and redundant activity with Zcchc11 in regulating pre-let-7 levels in embryonic stem cells (18) (Figure 4a). We tested whether this redundant activity was also shared for the ability to uridylate a subclass of mature miRNAs (Figure 4b). Consistent with previous work showing similar activity between the two proteins, we found Zcchc6 to have a similarly strong preference for let-7 guide and miR-10a guide over let-7 passenger and miR-26 as for Zcchc11 (Figure 4b). The full-length Zcchc11 is a 184 kDa non-canonical poly(A) polymerase that is highly conserved across vertebrates. Zcchc6 is slightly smaller and lacks domains at the N- and C- termini. Since analysis of the Flag-Zcchc11 purified protein by silver staining revealed that the Zcchc11-containing complexes used for *in vitro* uridylation assays (in Figures 1 and 2) might contain multiple associated proteins that could contribute to the selective miRNA uridylation activity we observed, we next analyzed the activity of recombinant Zcchc11 (r.Zcchc11) expressed and purified from bacteria (Figure 4d and e). Indeed isolated r.Zccch11 displayed the same preference for uridylating let-7 guide miRNAs with an overall activity comparable to that of the Flag-Zcchc11 purified from mammalian cells (Figure 4e), thereby demonstrating the sufficiency of Zcchc11 for this selective miRNA uridylation activity. Single-nucleotide resolution analysis of miRNA uridylation assays with r.Zcchc11 revealed a single band that corresponds to the mature miRNA with a two-nucleotide extension (Figure 4f). We also measured the relative binding of r.Zcchc11 to either the let-7 guide or let-7 passenger RNA sequences. Consistent with the preferential uridylation activity we observed, we found that purified Zcchc11 preferentially associates with let-7 guide RNA in an electromobility shift assay (EMSA) (Figure 4g).

### Zcchc6 and Zcchc11 expression is developmentally regulated

**Figure 5.**
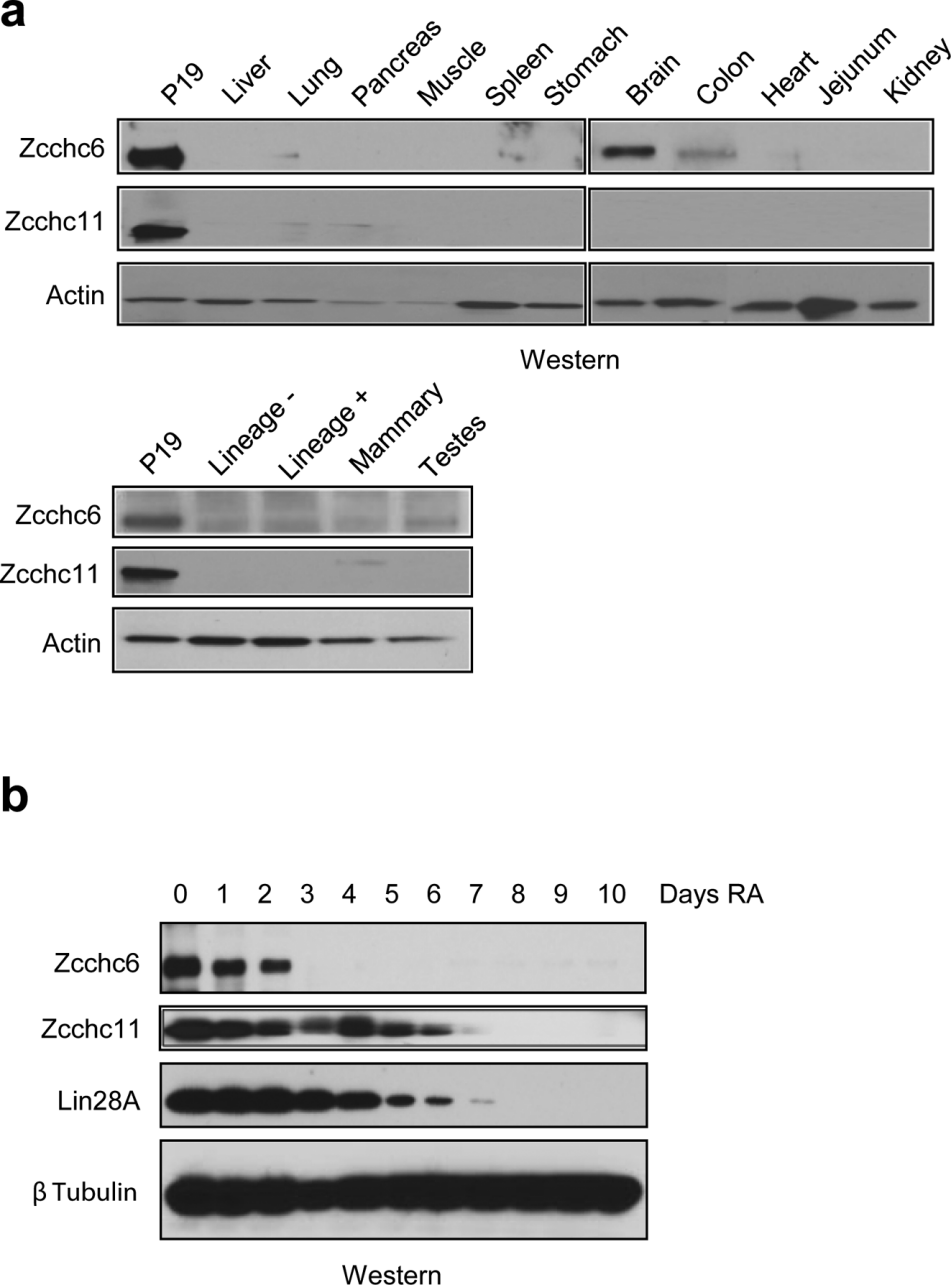
Zcchc11 and Zcchc6 are developmentally regulated TUTases. (**a**) Western blot analysis in extracts from adult mouse tissues. Zcchc11 and Zcchc6 are largely absent from differentiated tissues with the exception of Zcchc6 in the brain and colon. ‘Lineage –’ from fluorescently sorted hematopoietic cells lacking differentiation markers. ‘Lineage +’ from differentiated hematopoietic cells. (**b**) Retinoic acid (RA) mediated *in vitro* differentiation time course of the murine embryonal carcinoma cell line P19. Cells were cultured in the presence of RA for 10 days and analyzed for TUTase protein abundance. Lin28 serves as a differentiation positive control.

The extent of 3′ terminal uridylation of individual miRNAs is reported to be variable during cell differentiation, development and in different tissues (4,7). Considering this, we next examined the expression of Zcchc6 and Zcchc11 proteins in a panel of different adult mouse tissues where let-7 miRNAs are abundantly expressed. Interestingly, this analysis revealed that expression of both of these TUTases is undetectable in most adult tissues examined (Figure 5a). These results are consistent with the recently reported age-dependent expression of Zcchc11, with strongest expression observed in most organs at young ages (8). Extract from P19 embryonal carcinoma (EC) cells was used as positive control since we have previously reported an overlapping function for Zcchc6/11 in the Lin28-mediated control of let-7 biogenesis in these cells (18). This raised the possibility that expression of Zcchc6 and Zcchc11 is dynamically regulated during development and/or cell differentiation. To further investigate this, we treated P19 cells with retinoic acid and monitored Zcchc6/11 expression as cells differentiated over a time-course of several days. We found that while Zcchc6 and Zcchc11 protein was robustly detected in undifferentiated cells, the expression of these proteins was rapidly downregulated during cell differentiation. The temporal kinetics for changes in Zcchc11 closely mirrored the expression of Lin28A protein, whereas Zcchc6 seems to be more rapidly downregulated during the initial phase of cell differentiation (Figure 5b).

### Zcchc6/11 depletion does not affect miRNA abundance

**Figure 6.**
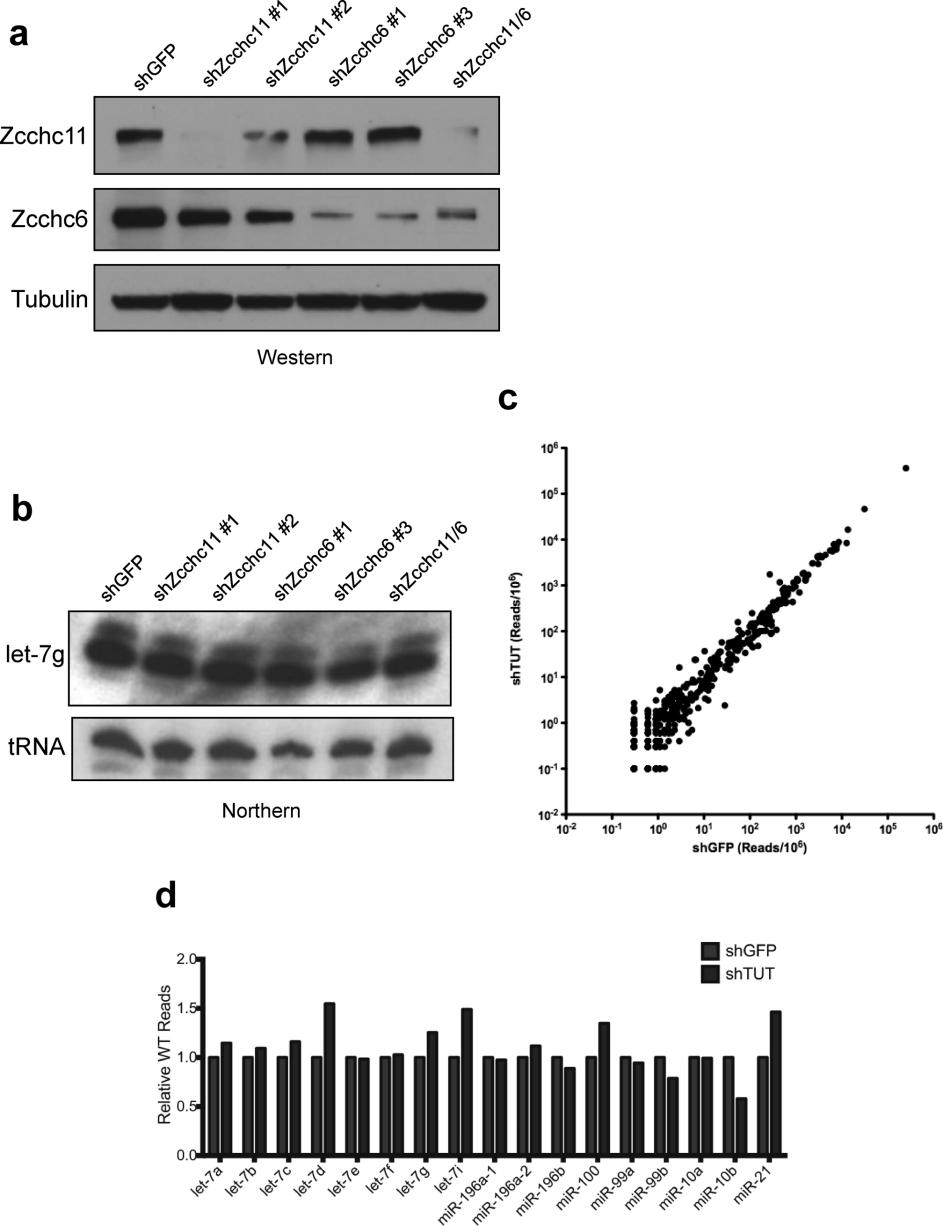
TUTase depletion does not affect miRNA levels. (**a**) Western blot analysis of Lentivirus-mediated knockdown of Zcchc11 and Zcchc6 in Hela cells. (**b**) Mature miRNA northern blot of let-7g levels in the indicated transduced Hela cell lines. Valyl tRNA serves as a loading control. (**c**) Total small RNA from shGFP and shZcchc6/shZcchc11 (shTUT) Hela cells was purified and cloned before sequencing. Each point represents a single miRNA species, and axes are scaled to reads/million (RPM). *R*^2^ >0.99. (**d**) The relative number of reads for WT (i.e. non-modified) motif-containing miRNAs is unchanged after TUTase depletion.

Our biochemical assays suggest that Zcchc11 and Zcchc6 may regulate the uridylation of a specific subset of miRNAs. To test this hypothesis, we examined the impact of Zcchc11 and Zcchc6 depletion on miRNA levels in cultured cells. Because both Zcchc11 and Zcchc6 function with Lin28 to regulate pre-let-7 in many cell types, we performed the following experiments in Hela cells, as they do not express either Lin28A or Lin28B, but express both TUTases (30). Hela cells were stably transduced with lentiviruses targeting Zcchc11, Zcchc6 or both genes together using different selectable markers (Figure 6a). Total RNA was purified from these stable cell lines and levels of let-7g were analyzed by northern blot. There was no detectable change in the abundance of let-7g, consistent with previous reports that showed no change in the steady-state levels of mature miRNAs after Zcchc11 depletion (6,8). Interestingly, the minor let-7g species detectable by northern blot did not change in either, suggesting that these bands may arise from cleavage heterogeneity rather than non-templated nucleotide addition (Figure 6b). To more comprehensively examine the overall levels and 3′ status of mature miRNAs in TUTase-depleted Hela cells, we cloned and sequenced small RNA libraries from control (shGFP) and Zcchc11/Zcchc6 double knockdown cells (shTUT). Total reads of all miRNAs irrespective of 3′ status were examined and remained largely unchanged (Figure 6c). The relative wild-type levels of predicted TUTase substrate miRNAs were also largely unchanged, with no changes larger than 2-fold in either direction (Figure 6d). These experiments indicate that depletion of Zcchc11 and Zcchc6 does not dramatically change the expression landscape of miRNAs in Hela cells, even those predicted as preferred substrates of these two TUTases.

### TUTase depletion causes selective loss of miRNA mono-uridylation and concomitant gain of mono-adenylation

**Figure 7.**
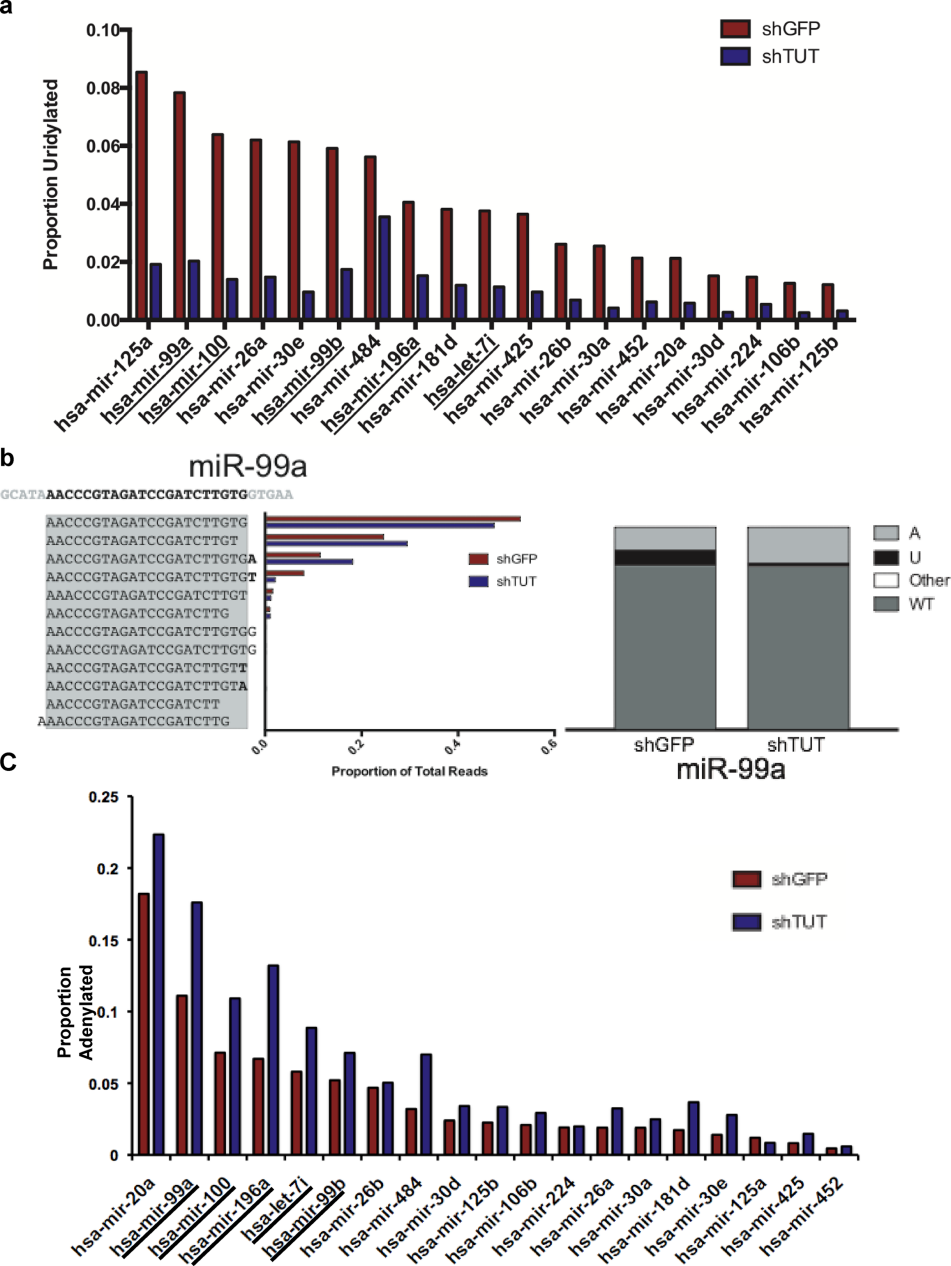
TUTase depletion causes selective loss of miRNA mono-uridylation and concomitant gain of mono-adenylation. (**a**) Rank order of the most highly uridylated 5p miRNAs were predicted to be TUTase targets. Color coding as in Figure 6 (d) (left) Distribution of proportional individual miRNA species for miR-99a. Genomic sequence is listed at top left, with the gray box representing the canonical mature miRNA sequence and the bold residues indicating non-templated nucleotides added after Dicer cleavage. (Right) Distribution of 3′ terminal residues for miR-99a in control and TUTase-depleted cells showing reads ending in non-templated adenosines (light gray), uridines (black), cytidines or guanosines (white, ‘other’) or with genomically encoded residues (dark gray, ‘WT’). (**c**) Rank order of miRNAs from Figure 7a sorted by relative 3′ adenylation status in shTUT cells.

To determine if the 3′ status of miRNAs predicted in this study are dramatically altered in the absence of Zcchc11 and Zcchc6, we compared their overall uridylation status with a large subset of mature miRNAs expressed in Hela cells. One major obstacle in studying mature miRNA uridylation is determining at which processing step non-templated nucleotides are added, since mature miRNAs can be produced from either side of a pre-miRNA hairpin. If mature miRNAs are derived from the 3′ arm of the precursor loop (3p-miRs), then uridylation can occur upstream of Dicer (15). These 3p-miRs may also undergo mature miRNA uridylation after Dicer cleavage or after strand selection and incorporation into RISC. Conversely, mature miRNAs derived from the 5′ arm of a precursor loop (5p-miRs) can only undergo uridylation after Dicer cleavage, either before or after strand selection and RISC incorporation. Furthermore, non-templated U addition is enriched for 3p-miRs and widespread pre-miRNA uridylation has been reported for several distinct miRNA families beyond let-7, complicating the analysis of 3p-miR uridylation (4,31). Fortuitously, all of the predicted miRNAs from our analysis are derived from the 5p arm of their precursor hairpins as determined by the relative read number in our experiments as well as by online databases such as miRbase (32). To examine the levels of uridylation for predicted TUTase substrate miRNAs, we compared the 3′ ends of these miRNAs to all other 5p-miRs so that we were exclusively observing mature miRNA uridylation rather than a combination of pre-miRNA and mature miRNA effects. For a more stringent comparison of 5p-miRs we examined only those that contained non-templated uridylated residues at a frequency of 10 reads per million (RPM) or more and made up no less than 1% of reads for a single miRNA in the shGFP sample. Of the 19 miRNAs that fall into this category in shGFP Hela cells, uridylation occurs between 1 and 8% and in all cases it is substantially reduced after Zcchc11 and Zcchc6 depletion (Figure 7a). Notably, several of the miRNAs predicted in our analysis appear in this group, making up 5 of the top 10 most-uridylated miRNAs (Figure 7a, underlined). Several predicted miRNAs fell just below the threshold criteria, including miR-196b, let-7e, miR-98 and let-7b (Supplementary Figure S1). Nevertheless the presence of multiple miRNAs predicted from our *in vitro* assays in the list of miRNAs with uridylation changes was statistically significant (*P* = 0.00296, Fisher's Exact test). In all instances, these miRNAs also underwent a significant loss of uridylation similar to those listed above (data not shown).

In addition to ranking the most uridylated miRNAs, we wished to examine the composition of the 3′ ends of this miRNA subclass. As shown in Figure 7b, miR-99a predominantly exists in its 22-nucleotide (nt) form, with a significant proportion being 21nt in length. The next most common species is the 22nt isoform extended by an untemplated adenosine residue, followed by the 22nt form extended by a single uridine residue. Reads containing a non-templated uridine residue are significantly reduced after TUTase depletion, however we observe an increase in adenylated reads in response to TUTase depletion as well. It may also be the case that the cleavage site for miR-99a production is shifted from the 22nt to the 21nt form after TUTase loss. This could be due to changes in 3′ pre-miRNA uridylation altering the Dicer cleavage site, or this could be due to other unexplored effects of mature miRNA uridylation.

To understand the 3′ modification landscape of modified miRNAs, we compared the terminal nucleotides of these miRNAs in shGFP and shTUT cells. Restricting our analysis to sequences that are present at 1 RPM or greater in shGFP cells, genomic reads of miR-99a make up 80% of all reads, with non-templated uridine residues contributing 8%, non-templated adenosines contributing 11% and the remainder coming from cytidine or guanine addition. Intriguingly, as uridylation levels are decreased after knockdown of Zcchc11 and Zcchc6, there is a concomitant increase in non-templated adenosine residues (Figure 7c) (*P* = 0.00244, Fisher's exact test). This phenomenon is found in nearly all predicted TUTase substrate miRNAs and is common in nearly all of the top uridylated miRNAs. The impact of this compensatory adenylation is unknown and it remains unclear which enzyme is responsible for this modification. These experiments together show that a select group of miRNAs undergo Zcchc11- and Zcchc6-dependent uridylation in cultured cells, and that half of the top miRNAs undergoing uridylation can be predicted by a simple sequence motif from *in vitro* studies. Furthermore, with the loss of the these two TUTases and the marks of their catalytic activity, non-templated adenylation often appears, which effectively retains a constant proportion of miRNA reads corresponding to genomic sequences.

### TUTase inhibition causes developmental defects and aberrant *Hox* gene expression in Zebrafish

**Figure 8.**
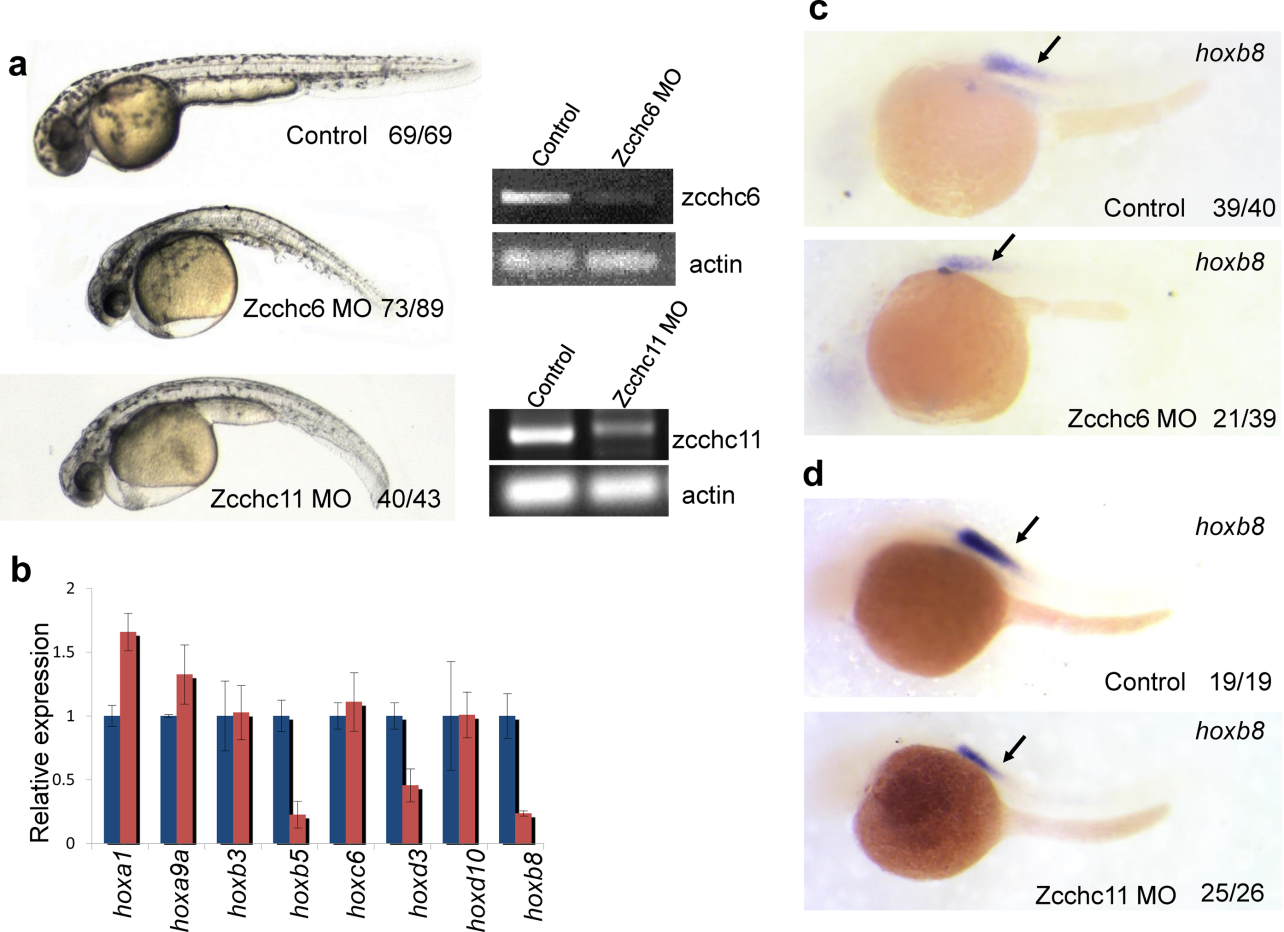
Inhibition of zebrafish *Zcchc6/11* genes results in improper *Hox* expression and embryonic development. (**a**) The *zcchc6* or *zcchc11* morphant embryos display multiple defects at 48 hpf, including degeneration of somites, failure of tail elongation, pericardial edema and developmental delay. RT-PCR was used to measure the relative knockdown of Zcchc6 and Zcchc11. PCR products were resolved using agarose gel electrophoresis and visualized by ethidium bromide staining (**b**) Quantitative PCR analysis (on 24-h embryos) shows the reduction of several *Hox* genes. Blue bars are control and red bars represent Zcchc11 MO (**c**and**d**) *Hoxb8b* was reduced in *Zcchc6* and *Zcchc11* morphant embryos at 24 hpf. The numbers indicated in the right hand corner of each picture: the number of affected embryos similar to what is shown (left) and the total number of observed embryos (right).

To examine whether Zcchc6/11 genes play a role in animal embryonic development, we turned to Zebrafish, which possesses Zcchc6/11 homologs (Supplementary Figure S2). Knocking down Zebrafish Zcchc6 gene by anti-sense morpholino resulted in a minor developmental delay by 24-h post fertilization (hpf) (data not shown). After 24 hpf, embryos demonstrated multiple developmental defects including developmental delay, degeneration of somites, failure of tail elongation and pericardial edema (Figure 8a). These defects continued to worsen, and the majority of fish died after 5 days post fertilization (dpf). Inhibition of Zcchc11 by morpholino resulted in the same developmental delay and defects (Figure 8a). In addition, the phenotypes following inhibition of each gene were verified with a second non-overlapping morpholino (Supplementary Figure S3c and d). More importantly, knockdown of Zcchc6/11 genes in p53 mutant embryos caused similar defects (Supplementary Figure S3b), supporting the hypothesis that the phenotypes observed are specifically due to inhibition of Zcchc6/11 genes and not, for example, apoptosis. These findings indicate that Zcchc6 and Zcchc11 are required for proper embryonic development. Next, we investigated whether Zcchc6/11 regulates *Hox* genes in embryos. To avoid potential complications, we analyzed *Hox* expression at 24–28 hpf, before the developmental delay and defects appear. We examined a subset of *Hox* genes by quantitative reverse transcription PCR (q.RT-PCR), including *hoxa1a, hoxa9a*, *hoxb3a*, *hoxb5a*, *hoxb8b*, *hoxc6a*, *hoxd3a* and *hoxad10a* in pools of control and Zcchc6-depleted embryos. Among them, *hoxb5a*, *hoxb8a* and *hoxd3a* were significantly downregulated (Figure 8b). Consistent with these q.RT-PCR results, RNA *in situ* analysis showed the reduced expression of *Hoxb8b*, but not *Hoxa9a*, in most of the morpholino-injected embryos (Figure 8c and d and Supplementary Figure S3a). This suggests that Zcchc6/11 regulates a group of *Hox* genes in zebrafish embryos.

## DISCUSSION

In the last several years, a number of activities have been credited to Zcchc11 and Zcchc6 including positive and negative regulation of pre-let-7 miRNAs, mediating toll-like receptor (TLR) signaling, uridylation of a small subset of mature miRNAs, and regulating the cell cycle independent of its catalytic activity (6,8,15–18,30,33–36). Our findings add to the existing catalog of TUTase substrate RNAs and are the first examples to our knowledge of a sequence-dependent RNA–protein interaction for either of these enzymes. This specific activity is reminiscent of the U6 TUTase (TUTase6/TUT1/PAPD2/Hs5), which adds an oligo-uridine tail exclusively to the U6 snRNA (37). Mature miRNA uridylation by Zcchc11 and Zcchc6 has been shown to disrupt target mRNA repression without causing a change in the steady-state levels of the miRNA (6,8). It remains unclear how only one or two non-templated nucleotides can disrupt targeting of mRNAs since the 3′ end of miRNAs is thought to be largely dispensable for repression by RISC (38). This question warrants further investigation and remains one of the most outstanding questions in the field.

Upon identifying a sequence motif that is necessary and sufficient for miRNA targeting by Zcchc11 and Zcchc6, we were surprised to find a striking enrichment for miRNAs that target Hox genes. The miR-10, miR-99/100 and miR-196 families are some of the most common Hox gene-targeting miRNAs yet described. They are expressed either in clusters containing let-7 family members or from within Hox gene clusters themselves, and are known to regulate numerous Hox genes—in many cases those located in a nearby genomic locus. Hox-targeting by these miRNAs is thought to repress anterior expression patterns and promote posterior development (29). One notable example is the ability for miR-196a to bind a perfectly complementary 22nt sequence in the 3′UTR of HoxB8, a rare case in mammals that causes cleavage of the transcript similar to the activity of an siRNA (38,39). Given the known ability for uridylation to attenuate miRNA repression of target genes, it is possible that temporal regulation of Hox-targeting miRNAs may play a role in embryonic patterning. Consistent with this we find that inhibition of Zcchc6 or Zcchc11 in zebrafish embryos leads to developmental arrest and dysregulated *Hox* gene expression. However we cannot exclude the possibility that the observed developmental phenotypes caused by TUTase depletion might be due to the dysregulation of additional RNAs. It will be important to identify the full catalog of Zcchc6 and Zcchc11 substrate RNAs during embryonic development. Related to this, a novel technique that enables the high-throughput sequencing of the 3′ end of mRNAs revealed widespread uridylation downstream of poly(A) tails in cultured human cells (40). Future work should uncover the functional relevance of these mRNA modifications and identify the enzymes responsible. Although a Zcchc11 knockout mouse has been described and survives to birth at Mendelian ratios, the redundancy between the two TUTases in mature miRNA uridylation and pre-let-7 turnover suggests a double knockout animal is required before robust phenotypes are observed (8).

Interestingly, miR-125 family members are expressed from some of these same genomic clusters but do not contain the predicted targeting sequence. Still, they are highly uridylated in cultured HeLa cells and undergo a robust decrease in uridylation after loss of Zcchc11 and Zcchc6, and this is particularly true for miR-125a which was the most highly uridylated mature miRNA in our study. It is tempting to speculate that the miR-125 family—the mammalian homolog of the *C. elegans* heterochronic miRNA lin-4-is also targeted by Zcchc11 and Zcchc6 due to its known role in development, yet the mechanism of this targeting remains unknown. Indeed, we found miR-26 family members to undergo TUTase-dependent uridylation as reported previously even in the absence of the sequence motif, indicating that there are other levels of specificity that remain unknown. Also worth noting is the similarity in the seed sequence between let-7 family miRNAs and miR-196a/b. Although they belong to distinct miRNA families, their seed sequences are different only in that they are shifted by one nucleotide, suggesting that there may be an overlap in target mRNAs for these two miRNA families.

Sequencing of small RNAs isolated from shTUT HeLa cells uncovered uridylated reads for many of the miRNAs predicted by our *in vitro* studies. One complication of this approach is the ambiguity that arises when the genomic sequence of a miRNA is such that nucleotides after the canonical 3′ end of 5p-miRNAs are thymidine rich. In these instances it is impossible to determine if a terminal uridine residue is the result of Dicer cleavage heterogeneity or is the result of non-templated nucleotide addition. In the case of miR-10a/b, which contain the TUTase sequence motif and are uridylated *in vitro*, we were nonetheless unable to rank it in our list of highly uridylated miRNAs because of a genomic thymidine residue after the 3′ end of the mature miRNA. Still, upon TUTase depletion this terminal nucleotide is dramatically reduced and the corresponding adenylated species is increased (Supplementary Figure S1). This situation is found for several of our predicted miRNAs suggesting that we may be underestimating the extent of 3′ uridylation (and in some cases adenylation) in our studies, yet we cannot rule out that this uridylation reduction is due to a change in Dicer processing.

A recent study described a role of GLD2/PAPD4/TUTase 2 in stabilizing a subset of mature miRNAs in cultured cells (9). GLD2 was shown previously to stabilize mature miR-122 by adding a single non-templated adenosine residue in mouse liver and mouse embryonic fibroblasts (MEFs), and the recent study extended that work to show stabilization of several other miRNAs by a similar mechanism (9,10). Interestingly, a subset of miRNAs that are responsive to GLD2-mediated adenylation are specified by sequences in their 3′ ends and include some but not all let-7 family members (e.g. let-7i, identified as TUTase-dependent in our study). The authors also found that miR-99a and miR-196a are stabilized by GLD2 despite lacking a 3′ GLD2 stabilizing sequence (9). The overlap between GLD2-sensitive miRNAs and TUTase-dependent miRNAs suggests that GLD2 may be the PAP responsible for compensatory adenylation in the absence of Zcchc11 and Zcchc6. This adenylation activity may stabilize miRNAs in the absence of a uridylation residue and maintain levels of functional miRNAs. Alternatively, there may be a miRNA sensing mechanism that maintains a constant population of unmodified miRNAs, thus replacing uridylated species with adenylated ones.

Our *in vitro* analysis is largely supported by our deep sequencing results but there are notable exceptions including the general absence of many uridylated mature let-7 family members. While uridylation is detected above background for let-7i, this is complicated by adjacent genomic thymidine residues for most let-7 species including let-7a/b/c/d/f/g. For example, reads of let-7b contain a 3′ terminal U roughly 15% of the time, and this decreases 3-fold after TUTase depletion. Correspondingly, 3′ adenylation makes up 18% of reads in control cells compared to over 26% in shTUT cells, following the trend of other predicted miRNAs such as miR-99a (Figure 7c and Supplementary Figure S1). Similar issues exist for several other predicted miRNAs and therefore may significantly underestimate the extent of 3′ uridylation and/or adenylation. Finally, our analysis focuses primarily on 5p-miRs as all of our predicted substrate miRNAs are fortuitously derived from the 5′ arm of miRNA precursors. Many 3p-miRs were found to be significantly uridylated in our sequencing study (data not shown) but separating this mark from pre-miRNA effects is impossible at this time. Therefore there are likely at least a few 3p-miRNAs that are subjected to Zcchc11- and Zcchc6-dependent terminal uridylation. Elucidating the full panoply of modified miRNAs and understanding the consequences of this activity are exciting questions in this growing field and warrant further investigation as an insight to an important form of gene regulation.

## ACCESSION NUMBERS

Small RNA sequencing data–Gene Expression Omnibus (GEO), accession number: (GSE52575).

## SUPPLEMENTARY DATA

Supplementary Data are available at NAR Online.

SUPPLEMENTARY DATA
