# Supplementary material for: Selective microRNA uridylation by Zcchc6 (TUT7) and Zcchc11 (TUT4)
Source: Nucleic Acids Res. 2014 Sep 15;42(18):11777–91. doi: 10.1093/nar/gku805 (PMC4191393; doi:10.1093/nar/gku805)
Supplement: SUPPLEMENTARY DATA [file supp_42_18_11777__index.html]

Selective microRNA uridylation by Zcchc6 (TUT7) and Zcchc11 (TUT4) — SUPPLEMENTARY DATA 

# Selective microRNA uridylation by Zcchc6 (TUT7) and Zcchc11 (TUT4)

## SUPPLEMENTARY DATA

**Files in this Data Supplement:**

- SUPPLEMENTARY DATA
