# Supplementary material for: Selective microRNA uridylation by Zcchc6 (TUT7) and Zcchc11 (TUT4)
Source: Nucleic Acids Res. 2014 Sep 15;42(18):11777–91. doi: 10.1093/nar/gku805 (PMC4191393; doi:10.1093/nar/gku805)
Supplement: SUPPLEMENTARY DATA [file supp_gku805_nar-03440-r-2013-File011.pdf]

Legends

Supplementary Figure 1: TUTase depletion causes selective loss of miRNA mono-uridylation and concomitant gain of mono-adenylation. Distribution of proportional individual miRNA species for indicated miRNAs. Genomic sequence is listed at top left, with the gray box representing the canonical mature miRNA sequence and the bold residues indicating non-templated nucleotides added after Dicer cleavage.

Supplementary Figure 2: Sequence comparison of zebrafish and human *Zcchc6* and *Zcchc11* proteins. Predicted partial reference sequences, ENSDARG00000074645 for *zcchc6* and ENSDARG00000070271 for *zcchc11*, were annotated in zebrafish genome (http://www.wensemble.org/Danio_rerio/gene/). TBLASTN analysis using human zcchc6 or zcchc11 protein sequences against various zebrafish nucleotide sequence databases, such as EST, WGS contigs, TSA, and nr, identified the same sequences as the above annotated sequences. **(a-d)** Alignment of zebrafish and human Zcchc6/11 proteins. Identical amino acids and conserved amino acids (+) are listed. **(a-b)** Zcchc6; **(c-d)** Zcchc11. C2H2 domain **(a and c)** and catalytic domain **(b and d)** are highlighted in blue. Note: zcchc6 MO was designed to block the splicing of C2H2 domain containing exon and zcchc6 MO-2 was to block the exon encoding active domain. Zcchc11 MO and MO-2 were also designed to block the splicing of exons containing C2H2 domain and active domain, respectively.

Supplementary Figure 3: Verification of *zcchc6* or *zcchc11* morphant phenotypes in a p53 mutant background and with a second morpholino. (a) *hoxa9a* staining was not affected in *zcchc6* morphant embryos. **(b)** Morpholino-mediated knockdown of *zcchc6* or *zcchc11* genes in p53 mutant embryos resulted in similar defects as seen in wildtype embryos. RT-PCR was used to measure relative Zcchc6 and Zcchc11 levels and the PCR products were analyzed by agarose gel electrophoresis and ethidium bromide staining **(c-d)** A second morpholino against *zcchc6* or *zcchc11* caused reduction of *hoxb8b* in embryos.

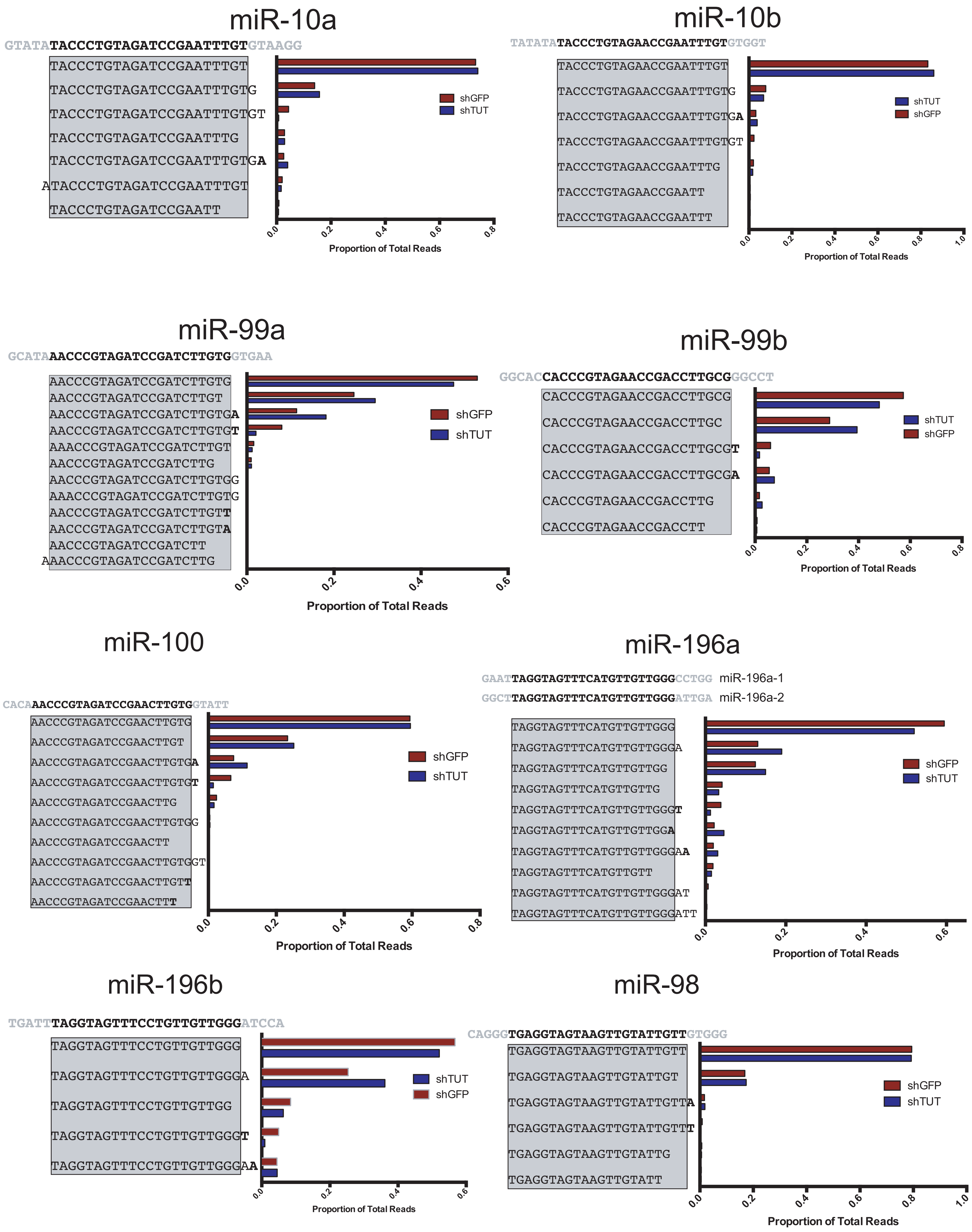
miR-10a
GTATATACCCTGTAGATCCGAATTTGTGTAAGG
TACCCTGTAGATCCGAATTTGT
TACCCTGTAGATCCGAATTTGTG
TACCCTGTAGATCCGAATTTGTGT
TACCCTGTAGATCCGAATTTG
TACCCTGTAGATCCGAATTTGTGA
ATACCCTGTAGATCCGAATTTGT
TACCCTGTAGATCCGAATT
shGFP
shTUT
0.0 0.2 0.4 0.6 0.8
Proportion of Total Reads
miR-10b
TATATATACCCTGTAGAACCGAATTTGTGTGGT
TACCCTGTAGAACCGAATTTGT
TACCCTGTAGAACCGAATTTGTG
TACCCTGTAGAACCGAATTTGTGA
TACCCTGTAGAACCGAATTTGTGT
TACCCTGTAGAACCGAATTTG
TACCCTGTAGAACCGAATT
TACCCTGTAGAACCGAATTT
shTUT
shGFP
0.0 0.2 0.4 0.6 0.8 1.0
Proportion of Total Reads
miR-99a
GCATAAACCCGTAGATCCGATCTTGTGGTGAA
AACCCGTAGATCCGATCTTGTG
AACCCGTAGATCCGATCTTGT
AACCCGTAGATCCGATCTTGTGA
AACCCGTAGATCCGATCTTGTGT
AAACCCGTAGATCCGATCTTGT
AACCCGTAGATCCGATCTTG
AACCCGTAGATCCGATCTTGTGG
AAACCCGTAGATCCGATCTTGTG
AACCCGTAGATCCGATCTTGTT
AACCCGTAGATCCGATCTTGTA
AACCCGTAGATCCGATCTT
AAACCCGTAGATCCGATCTTG
shGFP
shTUT
0.0 0.2 0.4 0.6
Proportion of Total Reads
miR-99b
GGCACCACCCGTAGAACCGACCTTGCGGGCCT
CACCCGTAGAACCGACCTTGCG
CACCCGTAGAACCGACCTTGC
CACCCGTAGAACCGACCTTGCGT
CACCCGTAGAACCGACCTTGCGA
CACCCGTAGAACCGACCTTG
CACCCGTAGAACCGACCTT
shTUT
shGFP
0.0 0.2 0.4 0.6 0.8
Proportion of Total Reads
miR-100
CACAAACCCGTAGATCCGAACTTGTGGTATT
AACCCGTAGATCCGAACTTGTG
AACCCGTAGATCCGAACTTGT
AACCCGTAGATCCGAACTTGTGA
AACCCGTAGATCCGAACTTGTGT
AACCCGTAGATCCGAACTTG
AACCCGTAGATCCGAACTTGTGG
AACCCGTAGATCCGAACTT
AACCCGTAGATCCGAACTTGTGGT
AACCCGTAGATCCGAACTTGTT
AACCCGTAGATCCGAACTTT
shGFP
shTUT
0.0 0.2 0.4 0.6 0.8
Proportion of Total Reads
miR-196a
GAATTAGGTAGTTTCATGTTGTTGGGCCTGG miR-196a-1
GGCTTAGGTAGTTTCATGTTGTTGGGATTGA miR-196a-2
TAGGTAGTTTCATGTTGTTGGG
TAGGTAGTTTCATGTTGTTGGGA
TAGGTAGTTTCATGTTGTTGG
TAGGTAGTTTCATGTTGTTG
TAGGTAGTTTCATGTTGTTGGGT
TAGGTAGTTTCATGTTGTTGGA
TAGGTAGTTTCATGTTGTTGGGAA
TAGGTAGTTTCATGTTGTT
TAGGTAGTTTCATGTTGTTGGGAT
TAGGTAGTTTCATGTTGTTGGGATT
shGFP
shTUT
0.0 0.2 0.4 0.6
Proportion of Total Reads
miR-196b
TGATTTAGGTAGTTTCCTGTTGTTGGGATCCA
TAGGTAGTTTCCTGTTGTTGGG
TAGGTAGTTTCCTGTTGTTGGGA
TAGGTAGTTTCCTGTTGTTGG
TAGGTAGTTTCCTGTTGTTGGGT
TAGGTAGTTTCCTGTTGTTGGGAA
shTUT
shGFP
0.0 0.2 0.4 0.6
Proportion of Total Reads
miR-98
CAGGGTGAGGTAGTAAGTTGTATTGTTGTGGG
TGAGGTAGTAAGTTGTATTGTT
TGAGGTAGTAAGTTGTATTGT
TGAGGTAGTAAGTTGTATTGTTA
TGAGGTAGTAAGTTGTATTGTTT
TGAGGTAGTAAGTTGTATTG
TGAGGTAGTAAGTTGTATT
shGFP
shTUT
0.0 0.2 0.4 0.6 0.8 1.0
Proportion of Total Reads

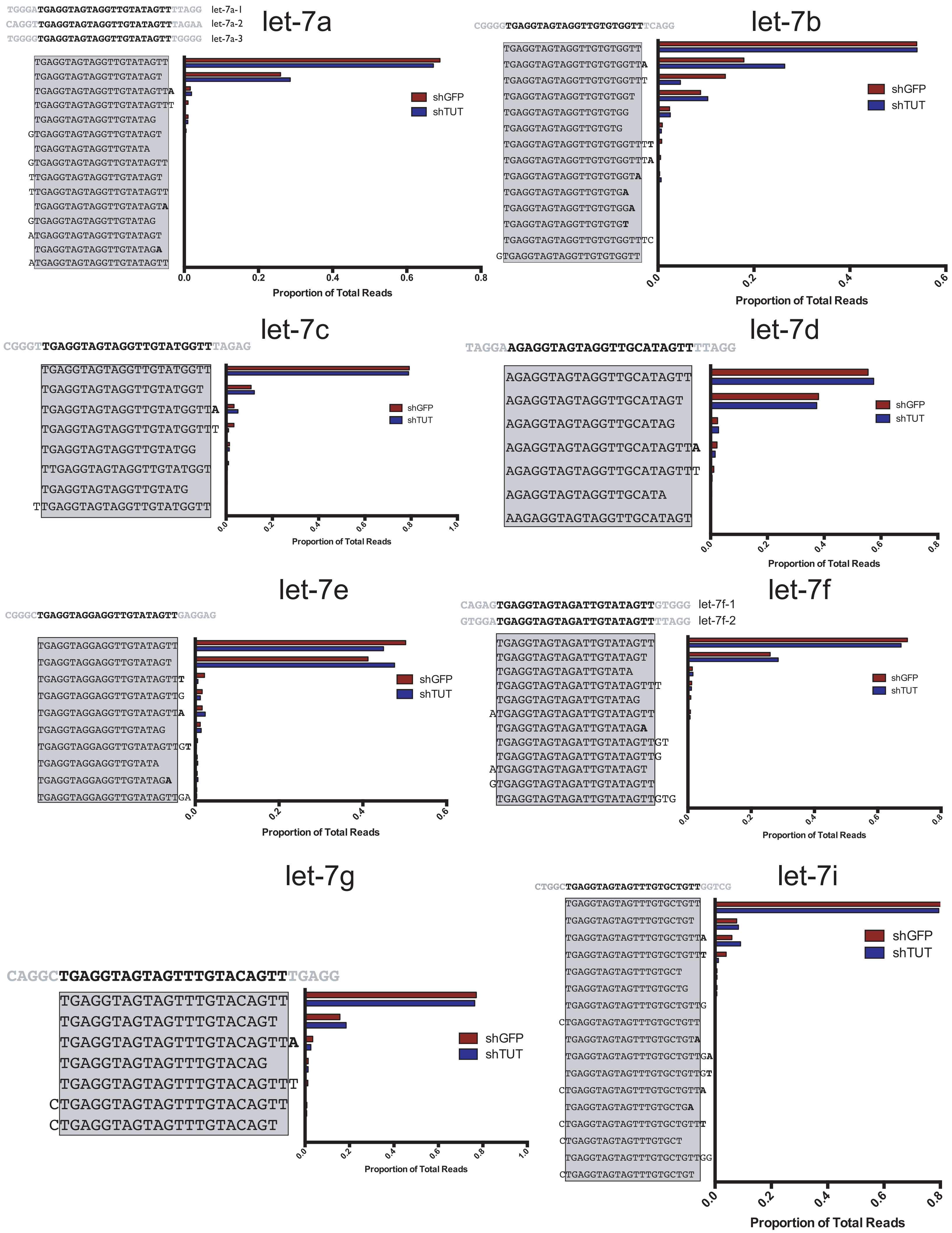
let-7a
TGGGATGAGGTAGTAGGTTGTATAGTTTTAGG let-7a-1
CAGGTTGAGGTAGTAGGTTGTATAGTTTAGAA let-7a-2
TGGGGTGAGGTAGTAGGTTGTATAGTTTGGGG let-7a-3
TGAGGTAGTAGGTTGTATAGTT
TGAGGTAGTAGGTTGTATAGT
TGAGGTAGTAGGTTGTATAGTTA
TGAGGTAGTAGGTTGTATAGTTT
TGAGGTAGTAGGTTGTATAG
GTGAGGTAGTAGGTTGTATAGT
TGAGGTAGTAGGTTGTATA
GTGAGGTAGTAGGTTGTATAGTT
TTGAGGTAGTAGGTTGTATAGT
TTGAGGTAGTAGGTTGTATAGTT
TGAGGTAGTAGGTTGTATAGTA
GTGAGGTAGTAGGTTGTATAG
ATGAGGTAGTAGGTTGTATAGT
TGAGGTAGTAGGTTGTATAGA
ATGAGGTAGTAGGTTGTATAGTT
shGFP
shTUT
0.0
0.2
0.4
0.6
0.8
Proportion of Total Reads
let-7b
CGGGGTGAGGTAGTAGGTTGTGTGGTTTCAGG
TGAGGTAGTAGGTTGTGTGGTT
TGAGGTAGTAGGTTGTGTGGTTA
TGAGGTAGTAGGTTGTGTGGTTT
TGAGGTAGTAGGTTGTGTGGT
TGAGGTAGTAGGTTGTGTGG
TGAGGTAGTAGGTTGTGTG
TGAGGTAGTAGGTTGTGTGGTTTT
TGAGGTAGTAGGTTGTGTGGTTTA
TGAGGTAGTAGGTTGTGTGGTA
TGAGGTAGTAGGTTGTGTGA
TGAGGTAGTAGGTTGTGTGGA
TGAGGTAGTAGGTTGTGTGT
TGAGGTAGTAGGTTGTGTGGTTTC
GTGAGGTAGTAGGTTGTGTGGTT
shGFP
shTUT
0.0
0.2
0.4
0.6
Proportion of Total Reads
let-7c
CGGGTTGAGGTAGTAGGTTGTATGGTTTAGAG
TGAGGTAGTAGGTTGTATGGTT
TGAGGTAGTAGGTTGTATGGT
TGAGGTAGTAGGTTGTATGGTTA
TGAGGTAGTAGGTTGTATGGTTT
TGAGGTAGTAGGTTGTATGG
TTGAGGTAGTAGGTTGTATGGT
TGAGGTAGTAGGTTGTATG
TTGAGGTAGTAGGTTGTATGGTT
shGFP
shTUT
0.0
0.2
0.4
0.6
0.8
1.0
Proportion of Total Reads
let-7d
TAGGAAGAGGTAGTAGGTTGCATAGTTTTAGG
AGAGGTAGTAGGTTGCATAGTT
AGAGGTAGTAGGTTGCATAGT
AGAGGTAGTAGGTTGCATAG
AGAGGTAGTAGGTTGCATAGTTA
AGAGGTAGTAGGTTGCATAGTTT
AGAGGTAGTAGGTTGCATA
AAGAGGTAGTAGGTTGCATAGT
shGFP
shTUT
0.0
0.2
0.4
0.6
0.8
Proportion of Total Reads
let-7e
CGGGCTGAGGTAGGAGGTTGTATAGTTGAGGAG
TGAGGTAGGAGGTTGTATAGTT
TGAGGTAGGAGGTTGTATAGT
TGAGGTAGGAGGTTGTATAGTTT
TGAGGTAGGAGGTTGTATAGTTG
TGAGGTAGGAGGTTGTATAGTTA
TGAGGTAGGAGGTTGTATAG
TGAGGTAGGAGGTTGTATAGTTGT
TGAGGTAGGAGGTTGTATA
TGAGGTAGGAGGTTGTATAGA
TGAGGTAGGAGGTTGTATAGTTGA
shGFP
shTUT
0.0
0.2
0.4
0.6
Proportion of Total Reads
let-7f
CAGAGTGAGGTAGTAGATTGTATAGTTGTGGG let-7f-1
GTGGATGAGGTAGTAGATTGTATAGTTTTAGG let-7f-2
TGAGGTAGTAGATTGTATAGTT
TGAGGTAGTAGATTGTATAGT
TGAGGTAGTAGATTGTATA
TGAGGTAGTAGATTGTATAGTTT
TGAGGTAGTAGATTGTATAG
ATGAGGTAGTAGATTGTATAGTT
TGAGGTAGTAGATTGTATAGA
TGAGGTAGTAGATTGTATAGTTGT
TGAGGTAGTAGATTGTATAGTTG
ATGAGGTAGTAGATTGTATAGT
GTGAGGTAGTAGATTGTATAGTT
TGAGGTAGTAGATTGTATAGTTGTG
shGFP
shTUT
0.0
0.2
0.4
0.6
0.8
Proportion of Total Reads
let-7g
CAGGCTGAGGTAGTAGTTTGTACAGTTTGAGG
TGAGGTAGTAGTTTGTACAGTT
TGAGGTAGTAGTTTGTACAGT
TGAGGTAGTAGTTTGTACAGTTA
TGAGGTAGTAGTTTGTACAG
TGAGGTAGTAGTTTGTACAGTTT
CTGAGGTAGTAGTTTGTACAGTT
CTGAGGTAGTAGTTTGTACAGT
shGFP
shTUT
0.0
0.2
0.4
0.6
0.8
1.0
Proportion of Total Reads
let-7i
CTGGCTGAGGTAGTAGTTTGTGCTGTTGGTCG
TGAGGTAGTAGTTTGTGCTGTT
TGAGGTAGTAGTTTGTGCTGT
TGAGGTAGTAGTTTGTGCTGTTA
TGAGGTAGTAGTTTGTGCTGTTT
TGAGGTAGTAGTTTGTGCT
TGAGGTAGTAGTTTGTGCTG
TGAGGTAGTAGTTTGTGCTGTTG
CTGAGGTAGTAGTTTGTGCTGTT
TGAGGTAGTAGTTTGTGCTGTA
TGAGGTAGTAGTTTGTGCTGTTGA
TGAGGTAGTAGTTTGTGCTGTTGT
CTGAGGTAGTAGTTTGTGCTGTTA
TGAGGTAGTAGTTTGTGCTGA
CTGAGGTAGTAGTTTGTGCTGTTT
CTGAGGTAGTAGTTTGTGCT
TGAGGTAGTAGTTTGTGCTGTTGG
CTGAGGTAGTAGTTTGTGCTGT
shGFP
shTUT
0.0
0.2
0.4
0.6
0.8
Proportion of Total Reads

a

```
Danio 1     KFRCDKCKVFCEDISPALSHIRERSHRKKAKELQKLALLLSIPPPAKPHCLSVSSALESI  60
            K+ C  C V  E I+ A  HI+E+ H+K  KE Q+  LL ++PPP      +V  A++ +
Hom   243   KYTCRLCDVLIESIAFAHKHIKEKRHKKNIKEKQEEELLTTLPPPTPSQINAVGIAIDKV  302

Danio 61    VTEFGLNDEDLKQRQNILALIEKVLHPVLPECQFRLYGSSSTKFGFKDSDVNIDVKFPSH  120
            V EFGL++E+L+QR  I  ++E V    LP+C  RLYGSS ++ GFK+SDVNID++FP+
Hom   303   VQEFGLHNENLEQRLEIKRIMENVFQHKLPDCSLRLYGSSCSRLGFKNSDVNIDIQFPAI  362

Danio 121   FQHPDILLAAQEHLSKSSLFDSVEGDFHRRMPVVVCKEKASGLICKVSAGNESACLTTAY  180
               PD+LL  QE L  S  F  V+ DFH R+PVVVC+EK SGL+CKVSAGNE+ACLTT +
Hom   363   MSQPDVLLLVQECLKNSDSFIDVDADFHARVPVVVCREKQSGLLCKVSAGNENACLTTKH  422

Danio 181   LSEMADLEPQLVPLVICFRYWAKICCVDQMEEGGLPSYCFALMVISFLQRRKEPILPTYL  240
            L+ +  LEP+LVPLVI FRYWAK+C +D+ EEGGLP Y FALM I FLQ+RKEP+LP YL
Hom   423   LTALGKLEPKLVPLVIAFRYWAKLCSIDRPEEGGLPPYVFALMAIFFLQQRKEPLLPVYL  482

Danio 241   ESM----------------------------------------AP-------------LA  247
             S                                         AP             L
Hom   483   GSWIEGFSLSKLGNFNLQDIEKDVVIWEHTDSAAGDTGITKEEAPRETPIKRGQVSLILD  542

Danio 248   FKSRSPVLLGKLWIELLRYYALEFQIPEKIISVRTNGELWRDLKDWPKKRIAIEDPFTVQ  307
             K +  V +G+LW+ELLR+YALEF + + +IS+R    + R+LKDWPKKRIAIEDP++V+
Hom   543   VKHQPSVPVGQLWVELLRFYALEFNLADLVISIRVKELVSRELKDWPKKRIAIEDPYSVK  602

Danio 308   RNVARTLNSQTMFDYLLHCLKTTYKYFASPSKSTAGKM----------STSSRSVSVNHA  357
            RNVARTLNSQ +F+Y+LHCL+TTYKYFA P K T   +          S  S+ V  +H
Hom   603   RNVARTLNSQPVFEYILHCLRTTYKYFALPHKITKSSLLKPLNAITCISEHSKEVINHHP  662

Danio 358   DSVKKSAKVSSEGL-NGPRVKSS  379
            D   K  K+ +  L  GP   SS
Hom   663   DVQTKDDKLKNSVLAQGPGATSS  685
```

b

```
Danio 501    DSESFHYVFNKRFFTDGKSPVQICGVCKSDGHLKQDCPEDFQRVELERLPPMTRDFQKIL  560
             D   F Y F+K  FT GKSP  +C +CK +GHLK+DCPEDF+R++LE LPP+T  F  IL
Hom   942    DQSDFFYEFSKLIFTKGKSPTVVCSLCKREGHLKKDCPEDFKRIQLEPLPPLTPKFLNIL  1001

Danio 561    SDVCEQCYRDFAPDDVELKVREHILQDFESFLRCQVPGKGAKLVLFGSSKNGFGFKQSDL  620
               VC QCY+DF+P   +E + REHI Q+ ESF+R   PG   KL LFGSSKNGFGFKQSDL
Hom   1002   DQVCIQCYKDFSPTIIEDQAREHIRQNLESFIRQDFPG--TKLSLFGSSKNGFGFKQSDL  1059

Danio 621    DICTLLNSHCRVNGLDSMAIIESLAKALRKHHGLRNILPITTAKVPIVKFYHTKTGLEGD  680
             D+C  +N      GLD +  IE LA+ LRKH GLRNILPITTAKVPIVKF+H ++GLE D
Hom   1060   DVCMTINGLETAEGLDCVRTIEELARVLRKHSGLRNILPITTAKVPIVKFFHLRSGLEVD  1119

Danio 681    ISLYNTLTLHDTLCLIISSLTSNFNIQMLNFLFCLHIKRSNIKMSSQMNISPMFIYNIKI  740
             ISLYNTL LH+T  L   S   +  ++ L +   +  K  +I  +S+ ++S  + Y + +
Hom   1120   ISLYNTLALHNTRLLSAYSAI-DPRVKYLCYTMKVFTKMCDIGDASRGSLS-SYAYTLMV  1177

Danio 741    TEFKHRRMPIIKLVCLQIYDGKQKPVVPVDGWDVYFFKDLKNLHRHWPEYKKNRESVGEL  800
               F  +R P +  V  +IY G++KP + VDGW++YFF  +  L  +W E  KN ESVG+L
Hom   1178   LYFLQQRNPPVIPVLQEIYKGEKKPEIFVDGWNIYFFDQIDELPTYWSECGKNTESVGQL  1237

Danio 801    WLGLLQFYTETFDFRESVICIRRKEPLSTFKKQWTSKHLAIEDPFDLSHNLGAGLSRRMA  860
             WLGLL+FYTE FDF+E VI IRRK  L+TFKKQWTSK++ IEDPFDL+HNLGAGLSR+M
Hom   1238   WLGLLRFYTEEFDFKEHVISIRRKSLLTTFKKQWTSKYIVIEDPFDLNHNLGAGLSRKMT  1297

Danio 861    SFIMKAFINARRVFGSP  877
             +FIMKAFIN RRVFG P
Hom   1298   NFIMKAFINGRRVFGIP  1314
```

Supplementary Figure 2

c

```
Danio 19    CSGMEDPKSPVKALKPSRSNAGKASSSKPQRESVKTPSRSKDVVPRVKEEAPGGRTRTD-   77
            C   + P SPVKA K + S A    ++  Q ++ K+P     V     E+A   + +++
Hom   96    CKAKKFPNSPVKAEKATISQAKSEKATSLQAKAEKSPKSPNSVK---AEKASSYQMKSEK   152

Danio 78    ---SPQEQARGKT---RRLTGRTNSGERGKGKPS------------------ATEARQQT   113
               SP E  +G +   + +  +T   +  GK  PS                  A +   ++
Hom   153   VPSSPAEAEKGPSLLLKDMRQKTELQQIGKKIPSSFTSVDKVNIEAVGGEKCALQNSPRS   212

Danio 114   QENKTAVRNSGPVKDGGATAEESSAHNNNTTTTTKDKMPGVEAGRQRVEEVVEVLNLTSD   173
            Q+ +T   N+G   D  +  E+ S   +       +K    E        V++   LT +
Hom   213   QKQQTCTDNTGDSDDSASGIEDVSDDLSKMKNDESNKENSSEMDYLENATVIDESALTPE   272

Danio 174   QQLGLKQAEERLQRDYIHRLLKPSPEYPNFQYLCKLCSVHVENIQGAHKHIKEKRHKKNI   233
            Q+LGLKQAEERL+RD+I RL K SPEY N +YLCKLC +H+ENIQGAHKHIKEKRHKKNI
Hom   273   QRLGLKQAEERLERDHIFRLEKRSPEYTNCRYLCKLCLIHIENIQGAHKHIKEKRHKKNI   332

Danio 234   MEKREENELRALPAPSPAQLRALDFAVLEAAELHGISEEDFALRQAVVLRMEGIIQKQLA   293
            +EK+EE+ELR+LP PSPA L AL  AV+E A+ HGI+++D  +RQ +V  M  +I   L
Hom   333   LEKQEESELRSLPPPSPAHLAALSVAVIELAKEHGITDDDLRVRQEIVEEMSKVITTFLP   392

Danio 294   ACSLRLYGSCLTRFAFKTSDVNIDVSYPSTMTQPDVLIQVLEILKNCVEFAEVESDFHAK   353
             CSLRLYGS LTRFA K+SDVNID+ +P  M  PD+LI+VL ILK  V + +VESDFHAK
Hom   393   ECSLRLYGSSLTRFALKSSDVNIDIKFPPKMNHPDLLIKVLGILKKNVLYVDVESDFHAK   452

Danio 354   VPVVFCRDEASGLMCKVSAGNDVACLTTNHLAALSRLEPRLVPLVLAFRYWANLCHIDCQ   413
            VPVV CRD  SGL+C+VSAGND+ACLTT+ L AL ++EP  +PLVLAFRYWA LC+ID Q
Hom   453   VPVVVCRDRKSGLLCRVSAGNDMACLTTDLLTALGKIEPVFIPLVLAFRYWAKLCYIDSQ   512

Danio 414   AEGGIPSYSLSLMVIFFLQQRYKPVLPVYLGHWIKGFEVKRVDEFHLTGVQSEVFVGWEH   473
             +GGIPSY  +LMV+FFLQQR  P+LP  LG WI+GF+ KR+D+F L G+  E FV WE
Hom   513   TDGGIPSYCFALMVMFFLQQRKPPLLPCLLGSWIEGFDPKRMDDFQLKGIVEEKFVKWEC   572

Danio 474   RPAPAGE---------GRGDGRSKPEPKTPVEKKEKKLEQTTGKSRLKLEEPVSASLGQL   524
              + A E          + D       KT  + +   +++  GKS L LE P   SLGQL
Hom   573   NSSSATEKNSIAEENKAKADQPKDDTKKTETDNQSNAMKEKHGKSPLALETPNRVSLGQL   632

Danio 525   WLELLRFYTLEFALEEHIISIRLKELLPRELKNWPRRRLAIEDPFALKRNVARSLNSQMV   584
            WLELL+FYTL+FALEE++I +R++++L RE KNWP+RR+AIEDPF++KRNVARSLNSQ+V
Hom   633   WLELLKFYTLDFALEEYVICVRIQDILTRENKNWPKRRIAIEDPFSVKRNVARSLNSQLV   692

Danio 585   FEYIQERFRTAYKYFACPQSR   605
            +EY+ ERFR AY+YFACPQ++
Hom   693   YEYVVERFRAAYRYFACPQTK   713
```

d

```
Danio 277   RQAVVLRMEGIIQKQL-AACSLRLYGSCLTRFAFKTSDVNIDVSY-----PSTMTQPDVL   330
            R+ +++ +E  IQK+      L L+GS    F F+ SD++I ++         +   +++
Hom   972   REQILIGLEKFIQKEYDEKARLCLFGSSKNGFGFRDSDLDICMTLEGHENAEKLNCKEII   1031

Danio 331   IQVLEILKNCVEFAEVESDFHAKVPVVFCRDEASGLMCKVSAGNDVACLTTNHLAALSRL   390
              + +ILK       +     AKVP+V      SGL   +S  N +A   T  LA  + +
Hom   1032  ENLAKILKRHPGLRNILPITTAKVPIVKFEHRRSGLEGDISLYNTLAQHNTRMLATYAAI   1091

Danio 391   EPRLVPLVLAFRYWANLCHIDCQAEGGIPSYSLSLMVIFFLQQRYKPVLPVYLGHWIKGF   450
            +PR+  L    + +A  C I   + G + SY+  LMV++FLQQR  PV+PV      + F
Hom   1092  DPRVQYLGYTMKVFAKRCDIGDASRGSLSSYAYILMVLYFLQQRKPPVIPVLQ----EIF   1147

Danio 451   EVKRVDEFHLTGVQSEVFVGWEHRPAPAGEGRGDGRSKPEPKTPVEKKEKKLEQTTGKSR   510
            + K++ +  + G  +  F                           E+ +K+L  + GK+
Hom   1148  DGKQIPQRMVDGWNAFFF------------------------DKTEELKKRLP-SLGKN-   1181

Danio 511   LKLEEPVSASLGQLWLELLRFYTLEFALEEHIISIRLKELLPRELKNWPRRRLAIEDPFA   570
                   + SLG+LWL LLRFYT EF  +E++ISIR K+LL    K W  + +AIEDPF
Hom   1182  -------TESLGELWLGLLRFYTEEFDFKEYVISIRQKKLLTTFEKQWTSKCIAIEDPFD   1234

Danio 571   LKRNVARSLNSQMVFEYIQERFRTAYKYFACP   602
            L  N+   ++ +M   +I + F    K F  P
Hom   1235  LNHNLGAGVSRKMT-NFIMKAFINGRKLFGTP   1265
```

Supplementary Figure 2

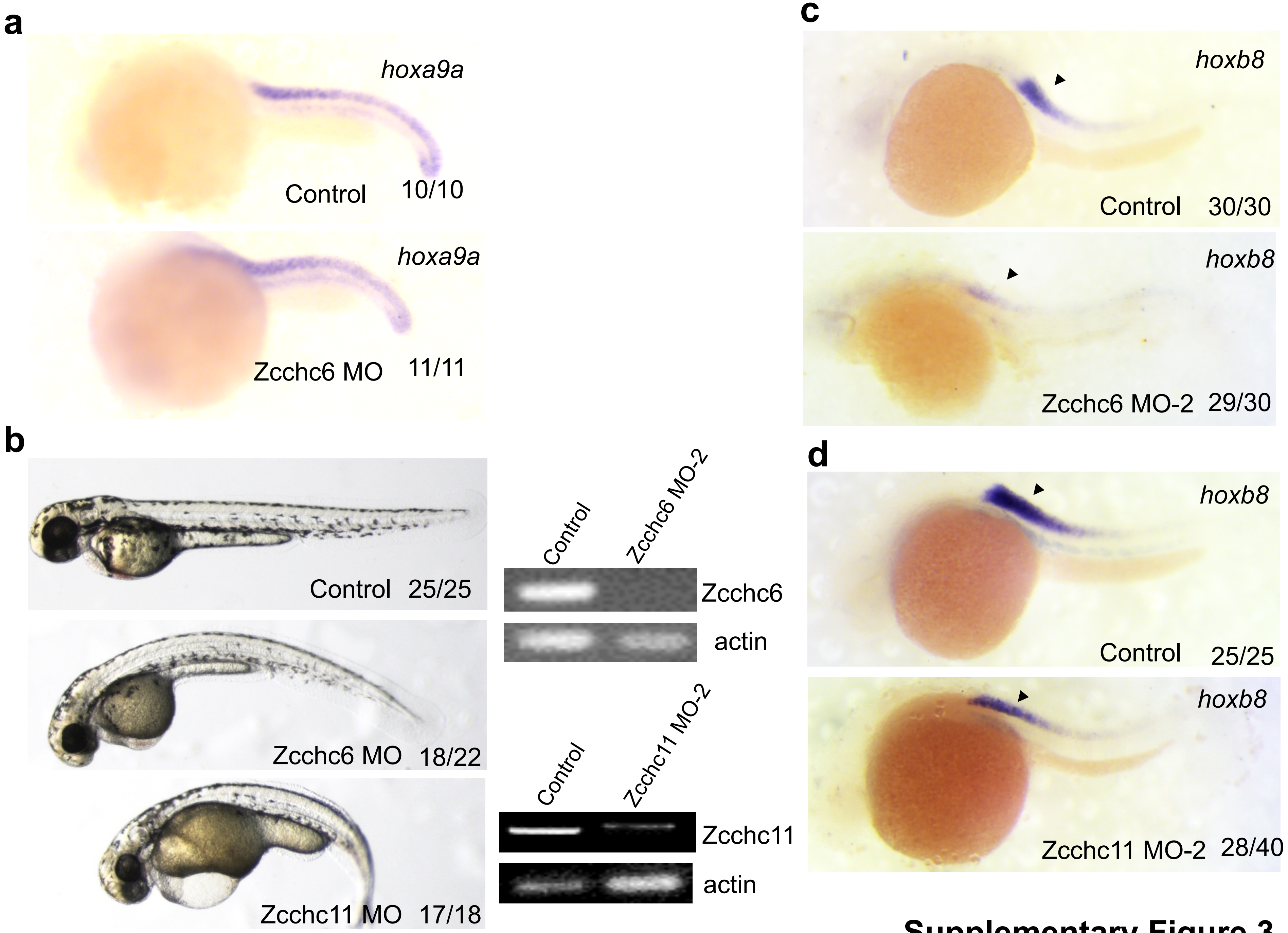

Supplementary Figure 3
